# Insights into the complex relationship between triglyceride glucose-waist height ratio index, mean arterial pressure, and cardiovascular disease: a nationwide prospective cohort study

**DOI:** 10.1186/s12933-025-02657-0

**Published:** 2025-02-28

**Authors:** Jie Xu, Dihui Cai, Yuheng Jiao, Yingying Liao, Yinyin Shen, Yunli Shen, Wei Han

**Affiliations:** 1 Department of Cardiology, Shanghai East Hospital, School of Medicine, Tongji University, Shanghai, China; 2 State Key Laboratory of Cardiology and Medical Innovation Center, Shanghai East Hospital, School of Medicine, Tongji University, Shanghai, China

**Keywords:** CHARLS, Triglyceride glucose-waist height ratio index, Mean arterial pressure, Cardiovascular disease, Mediation analysis

## Abstract

**Background:**

Both the triglyceride glucose-waist height ratio (TyG-WHtR) index and elevated arterial blood pressure are risk factors for cardiovascular disease (CVD). However, it is uncertain whether the TyG-WHtR index can increase the risk of CVD by influencing arterial blood pressure, and the extent to which the TyG-WHtR index may mediate the association between arterial blood pressure and CVD. The purpose of this study is to evaluate complex association of the TyG-WHtR index and mean arterial pressure (MAP) with CVD.

**Methods:**

All data in this study were obtained from the China Health and Retirement Longitudinal Study (CHARLS) free of CVD at baseline. CVD was defined as self-reporting heart disease and stroke. Cox proportional hazards model and restricted cubic spline (RCS) were used to analyze the association of the TyG-WHtR index and MAP with the risk of CVD. Time-dependent receiver operating characteristic (ROC) analysis was used to assess the predictive performance of TyG-WHtR, MAP for CVD. Four-way decomposition method explored the mediating effects of the TyG-WHtR index and MAP in CVD.

**Results:**

A total of 7976 participants were included in this study. The mean age of the participants was 58.68 ± 9.60 years, and 4263 (53.45%) were females. During a maximum follow-up of 7.0 years, 1326 (16.62%) people developed CVD. Both the TyG-WHtR index and MAP were signifcantly associated with CVD. The RCS regression analyses demonstrated a positive linear association of the TyG-WHtR index and MAP with the incidence of CVD. Compared with participants with TyG-WHtR < median and MAP < median, those with TyG-WHtR ≥ median and MAP ≥ median had the highest risk of CVD (HR 1.961; 95%CI 1.660–2.317). The combination of TyG-WHtR and MAP demonstrated incremental predictive utility over either biomarker alone, as evidenced by improvements in integrated discrimination improvement (IDI) and net reclassification improvement (NRI). While absolute predictive performance remained moderate. Increased MAP signifcantly mediated 52.43% of the associations between TyG-WHtR index and CVD, and increased TyG-WHtR index signifcantly mediated 83.40% of the associations between MAP and CVD.

**Conclusion:**

The combination of a higher TyG-WHtR index and a higher MAP was associated with the highest risk of CVD. The combined model of the TyG-WHtR index and MAP showed improved predictive ability, as indicated by IDI and NRI, although its overall predictive performance was still moderate. The MAP could partially mediate the association between TyG-WHtR index and CVD, as well as TyG-WHtR index could also partially mediate the association between MAP and CVD. These findings suggested that the combination of TyG-WHtR index and MAP helps identify populations at early risk of CVD and improve risk stratifcation.

**Graphical abstract:**

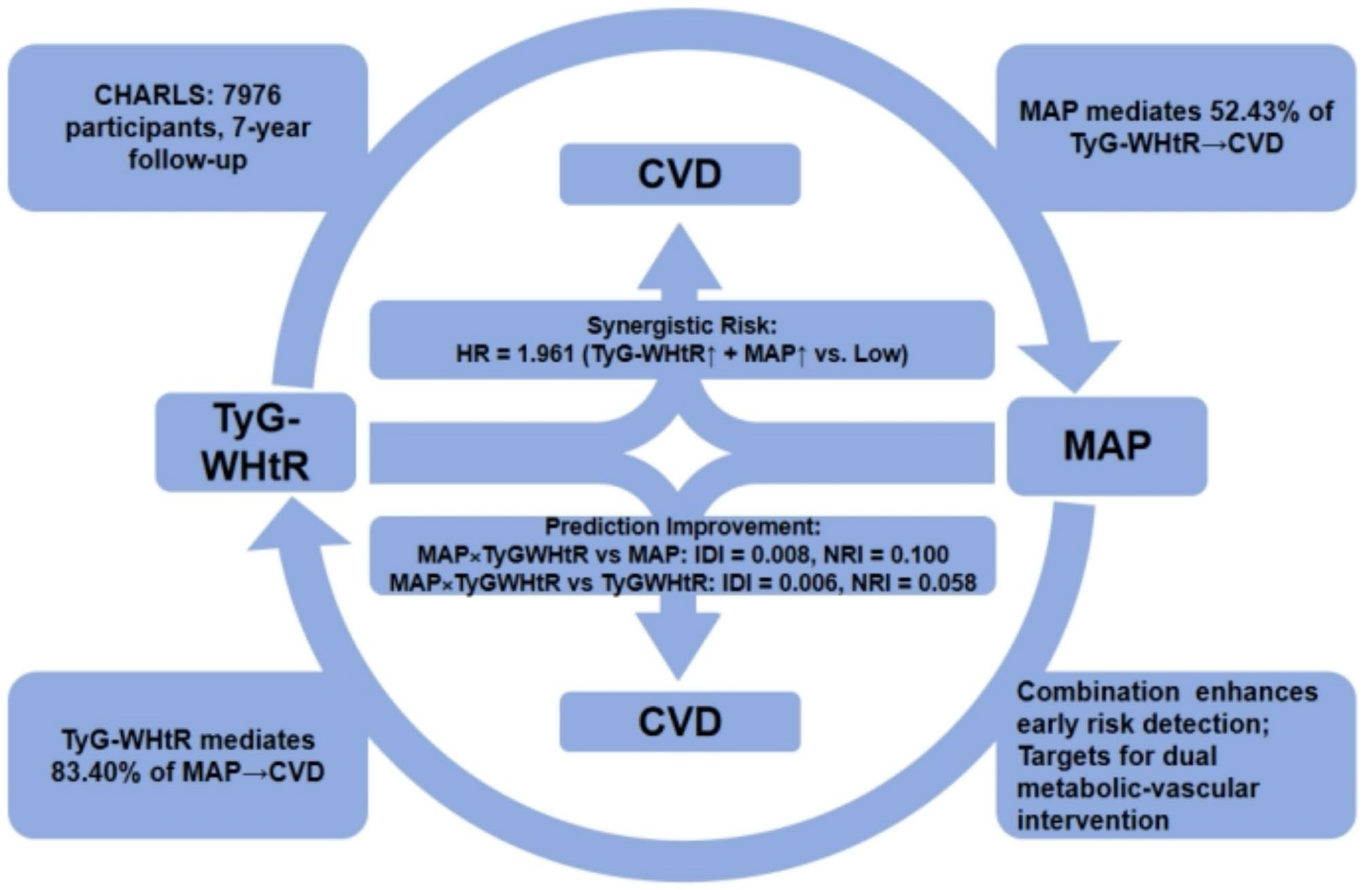

**Supplementary Information:**

The online version contains supplementary material available at 10.1186/s12933-025-02657-0.

## Introduction

Cardiovascular disease (CVD), encompassing heart disease and stroke, remains the leading cause of global mortality and a major contributor to rising healthcare costs [1, 2]. Atherosclerosis, the primary pathological driver of CVD, underlies clinical manifestations such as myocardial infarction and ischemic stroke [3]. With aging populations and lifestyle changes—including sedentary habits and poor dietary patterns—the global burden of CVD continues to escalate, positioning it as a critical public health priority [2, 4]. Early identification of modifiable risk factors and high-risk populations is essential for implementing targeted preventive strategies to mitigate this burden.

Hypertension is a pivotal risk factor for CVD [5], strongly associated with heart failure [6], chronic kidney disease [7, 8], and neurodegenerative disorders such as Alzheimer’s disease [9]. In this study, mean arterial pressure (MAP)—a hemodynamic marker reflecting average arterial pressure—serves as a representative measure of blood pressure. Elevated MAP is independently linked to increased CVD risk [10, 11], and its variability correlates with adverse vascular events, including wake-up stroke [12]. Insulin resistance (IR), a hallmark of metabolic syndrome, is also a significant contributor to CVD [13]. The triglyceride-glucose (TyG) index, a surrogate marker of IR, has emerged as a powerful predictor of CVD events [14–16]. Integration of TyG with waist-to-height ratio (WHtR) to form the TyG-WHtR index further enhances predictive accuracy, outperforming TyG alone in identifying high-risk subgroups [17, 18]. These findings underscore the critical roles of hypertension and IR in CVD pathogenesis.

Hypertensive populations exhibit a higher prevalence of IR compared to normotensive individuals [19]. Elevated blood pressure contributes to IR through multiple mechanisms, including chronic inflammation [20], renal dysfunction [21], and sympathetic nervous system activation [22, 23]. These pathways collectively impair insulin signaling and glucose metabolism, suggesting that IR may act as a mediating factor linking elevated blood pressure to CVD.

Conversely, IR exacerbates CVD risk by promoting abnormal glucose metabolism [19], arterial stiffness [24], and renal sodium-water retention [25], all of which contribute to increased blood pressure. This bidirectional relationship implies that elevated blood pressure may also mediate the association between IR and CVD. In this study, MAP serves as a representative measure of arterial blood pressure to explore the complex interplay between IR and CVD.

Despite the established roles of hypertension and IR in CVD, their potential mediating effects remain underexplored. This study aims to investigate the interrelationships between MAP, the TyG-WHtR index, and CVD, and elucidate the mediating roles of MAP and TyG-WHtR in CVD pathogenesis. By clarifying these mechanisms, our findings will be helpful for early identification of high-risk populations and personalized prevention strategies.

## Materials and methods

### Data source and study population

The data for the present investigation were obtained from the China Health and Retirement Longitudinal Study (CHARLS), a nationwide prospective cohort study. CHARLS employed a multistage stratified probability proportional-to-size sampling approach to recruit participants from both rural and urban regions covering 150 counties or districts across 28 provinces in China. The study conducted four regular biannual surveys between 2011 and 2018. Detailed descriptions of the study design and cohort profile have been previously reported in the literature [26]. The CHARLS study was conducted in full compliance with the principles of the Declaration of Helsinki and received ethical approval from the Institutional Review Board of Peking University (IRB00001052-11015). Prior to participation, all study subjects provided written informed consent. Rigorous and standardized training was provided to all fieldwork personnel, who subsequently administered face-to-face interviews utilizing standardized questionnaires [27]. In this study, participants interviewed from 2011 to 2012 were considered as the baseline, and then followed up in the years 2013, 2015, and 2018.

**Fig. 1:**
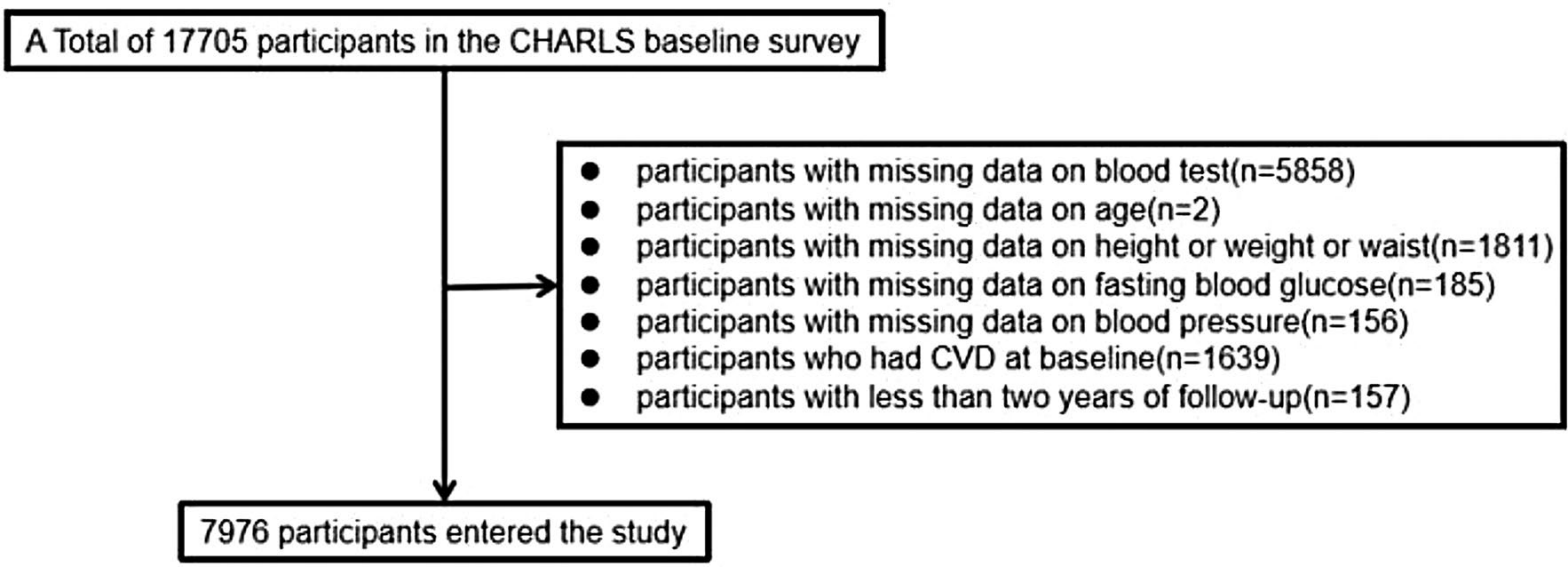
Flowchart of the study population

The flowchart (Fig. 1) delineates the inclusion and exclusion criteria of this study.Initially, 5858 participants were excluded due to the lack of blood sample test data, 2 participants were excluded due to missing age information, and 1811 participants were excluded due to the absence of height, weight, or waist circumference data. Additionally, 185 participants with missing fasting blood glucose data and 156 participants with missing blood pressure data were excluded. Furthermore, 1639 participants who had cardiovascular disease at baseline were also excluded. Finally, 157 participants with follow-up periods of less than two years were excluded from the analysis. Consequently, a total of 7976 participants were included in the analysis.

### Calculation of triglyceride glucose-waist height ratio index

WHtR = weight (cm) / height (cm)

TyG = ln [FPG (mg/dL) × TG (mg/dL) / 2]

TyG-WHtR = WHtR × TyG [28]

MAP= [SBP (mmHg) + 2 × DBP (mmHg)] / 3 [29]

FPG: fasting plasma glucose, TG: triglyceride, SBP: systolic blood pressure, DBP: diastolic blood pressure.

### CVD diagnosis

The primary outcome of interest in this study was the incidence of CVD during the follow-up period. CVD was defined as self-reporting heart disease and stroke. Specifically, the presence of heart disease was determined by participants’ affirmative response to the question, “Have you been diagnosed with heart attack, coronary heart disease, angina, congestive heart failure, or other heart problems by a doctor?” Similarly, the occurrence of stroke was ascertained through the question, “Have you been diagnosed with stroke by a doctor?” [30]. The research team of CHARLS implemented rigorous quality control measures for data recording and verification to ensure the reliability of the data [26]. The approach to CVD ascertainment in the current study was consistent with that of previous CHARLS-based investigations [31].

### Blood pressure measurement

In this study, the Omron™ HEM-7112 blood pressure monitor was used to measure blood pressure, manufactured by Omron Healthcare Co., Ltd. in Dalian [26]. The participant should be seated with their feet flat on the ground and their left arm resting comfortably, such as on a table, with the palm facing upwards. The participant should roll up their sleeve, unless they were wearing a short-sleeved shirt or a thin garment. The cuff size should be appropriate for the participant’s upper arm, ensuring direct contact with the skin, with the lower edge of the cuff approximately half an inch above the elbow. The air tube should be positioned facing the middle of the participant’s arm. The interviewer should then press the start button. The cuff would automatically inflate and, after displaying the systolic blood pressure, diastolic blood pressure, and pulse, it would deflate. The interviewer should record the systolic blood pressure, diastolic blood pressure, pulse, and the time of measurement. The interviewer should then wait 45–60 s before initiating the next measurement using a stopwatch or similar device.The blood pressure was measured three times on the left arm. The blood pressure values in baseline characteristics were the mean of these three repeated measurements.

### Blood sample collection

In the national survey, venous blood was collected by trained China CDC (Chinese Center for Disease Control and Prevention) staff, while non-blood biomarkers were collected by CHARLS enumerators. The collaboration with China CDC allowed for the collection of venous blood instead of dried blood spots, leveraging their nationwide network, trained staff, and experience in large-scale blood collection and processing. Three tubes of venous blood were collected from each respondent, with over 92% fasting. The first tube was used for a complete blood count test. After collection, these fresh venous blood samples were transported, at 4℃ temperature, to either local CDC laboratories or township level hospitals near the study sites. The second for plasma and buffy coat. The venous blood was processed and divided into these two components within the same timeframe as the complete blood count measurement. Transport to the local lab (if transport is required) was at 4℃. After the venous blood was separated into plasma and buffy coat, the plasma was then stored in three 0.5 mL cryovials and the buffy coat in a separate cryovial. These cryovials were then immediately stored frozen at − 20℃ and transported to the Chinese CDC in Beijing within 2 weeks where they were placed in a deep freezer and stored at -80℃ until assay at CMU laboratory. The third for the HbA1c assay. This 2 mL tube of whole blood was stored immediately and during shipment at 4℃, and transported to the China CDC in Beijing within 2 weeks, where it was placed at − 80℃ in a deep freezer for the HbA1c assay. The samples were processed and stored following strict protocols, with the majority of complete blood count tests conducted within 141 min of collection. The study was supported by the Chinese National Natural Science Foundation and the National Institute on Aging.

#### Anthropometric measurements

Height was measured using a portable stadiometer in participants capable of standing upright. In this study, Seca^TM^213 height monitor was used to measure height, manufactured by Seca Co., Ltd. in Hangzhou [26]. Participants were instructed to remove footwear and stand barefoot on the stadiometer platform with their back against the vertical. Heels were placed together, and feet were positioned at a 60-degree angle. The head was aligned to the Frankfurt plane (horizontal alignment of the tragus of the ear and the inferior orbital margin). A movable headboard was gently lowered to contact the vertex of the head, and the measurement was recorded to the nearest 0.1 cm.

Omron™ HN-286 weight scale was used to measure weight, manufactured by Krell Technology (Yangzhou) Co., Ltd [26]. Body weight was assessed using a calibrated digital floor scale for participants meeting inclusion criteria, excluding those self-reporting a weight > 150 kg or unable to stand independently. Prior to measurement, participants were asked to remove shoes, heavy outerwear, and empty pockets. The scale was placed on a flat, non-carpeted surface and calibrated to zero before each measurement. Participants stood motionless on the scale until a stable reading was obtained, recorded to the nearest 0.1 kg.

Waist circumference was measured at the level of the umbilicus using a non-elastic tape measure. Participants were required to stand with arms lifted to expose the waist area. After confirming the horizontal alignment of the tape around the waist, participants were instructed to exhale gently and hold their breath at the end of expiration. Measurements were taken at minimal respiration, ensuring the tape was snug but non-compressive. Two consecutive readings were averaged; if discrepancies exceeded 1.0 cm, a third measurement was obtained. All measurements were performed by trained staff following a standardized protocol.

### Data collection

I Demographic data: age, gender, education level, marital status.
II Body measurements: systolic blood pressure (SBP), diastolic blood pressure (DBP), waist circumference.
III Lifestyle data: smoking and drinking status, sleep problems.
IV Disease history: hypertension, diabetes, liver diseases, lung diseases, cancer, depression.
V Laboratory test data: hemoglobin (HGB), platelets (PLT), glycated hemoglobin A1c (HbA1C), fasting plasma glucose (FPG), blood urea nitrogen (BUN), serum creatinine (Scr), uric acid (UA), triglyceride (TG), total cholesterol (TC), high-density lipoprotein cholesterol (HDL-c), low-density lipoprotein cholesterol (LDL-c).

### Assessment of covariates

Those participants who reported a history of hypertension or were receiving any antihypertensive medication, as well as those with systolic blood pressure ≥ 130 mmHg or diastolic blood pressure ≥ 80 mmHg, were defined as hypertension [32]. Those who reported a history of diabetes or were receiving any hypoglycemic medication, as well as those with an fasting plasma glucose ≥ 7.0 mmol/L (126 mg/dL) or an HbA1c of ≥ 6.5% at baseline, were considered to have diabetes [33–34]. eGFR was calculated using the Chinese Modification of Diet in Renal Disease (C-MDRD) equation [35]. Depression was defined using the 10-item short form of the Center for Epidemiologic Studies Depression Scale (CESD10) [36]. Participants with a total score ≥ 10 were identified as exhibiting depressive symptoms. Sleep quality was judged by the question “My sleep in the last week was restless.“, which had four options: little or no time (< 1 day), some of the time (1–2 days), occasional or moderate time (3–4 days), and most or all of the time (5–7 days). Participants who answered ≥ 1 were identified as having sleep problem [37]. Other medical statuses were determined by self-report.

#### Handling of missing variables

The degree of missing data within this study is summarized in Table S1. To address missing values (0.57% of total data points) and maintain the most comprehensive sample size possible and thereby more accurately represent the actual conditions, we performed multiple imputation using chained equations algorithm [38]. All variables in the analysis were included in the imputation model. Five imputed datasets were generated with 10 iterations, and pooled estimates were obtained using Rubin’s rules [39].

#### Statistical analysis

At baseline, for continuous variables that displayed a normal distribution, statistics were described using means ± SD. For continuous variables that did not follow a normal distribution, the median and interquartile ranges were utilized for statistical description. Categorical variables were characterized by frequencies and percentages. We performed restrictive cubic spline (RCS) analyses to explore potential linear associations of the TyG-WHtR index and MAP with the incidence of CVD.

Survival curves were generated using the Kaplan-Meier (KM) method to estimate the cumulative incidence of CVD events over time. Participants were stratified by TyG-WHtR index and MAP categories. The log-rank test was employed to compare survival distributions between groups. To account for multiple comparisons when analyzing joint categories, the Bonferroni correction was applied by adjusting the significance level to α/6.

The TyG-WHtR index and MAP were grouped into four groups (Q1-Q4) based on quartiles (Additional file: Table S2, Table S3). Then, the combination of TyG-WHtR index and MAP was grouped into sixteen according to the quartiles of TyG-WHtR index and MAP. For continuous variables that displayed a normal distribution, statistics were described using means ± SD, and differences between groups were inferred using analysis of variance (ANOVA). For continuous variables that did not follow a normal distribution, the median and interquartile ranges were utilized for statistical description, and group-wise differences were examined using the Kruskal-Wallis H test. Categorical variables were characterized by frequencies and percentages, with intergroup differences assessed using the χ² test. The relationship between TyG-WHtR, MAP and the incidence of CVD was prospectively analyzed using multivariate Cox regression models (Additional file: Table S4, Table S5, Table S6).

Then, participants were grouped into four categories according to the joint assessment of the TyG-WHtR index and MAP (based on the median value, respectively), and Cox regression analysis was utilized for associations of the TyG-WHtR index and MAP with the incidence of CVD among the four categories. To assess the predictive performance of TyG-WHtR, MAP, and their product (MAP×TyG-WHtR) for CVD, we performed time-dependent ROC analysis, reporting the C-index with 95% confidence intervals (CIs). The IDI and NRI were further calculated to quantify incremental predictive value when combining TyG-WHtR with MAP, as recommended for risk model comparisons [40].

Mediation analyses were conducted to explore potential mechanistic pathways without assuming temporal precedence, not to infer causality. We applied the exploratory four-way decomposition method to disentangle direct, indirect, and interactive effects, acknowledging that temporal precedence cannot be established with cross-sectional data [41, 42]. Total effects were decomposed into four components: controlled direct effects, reference interaction, mediated interaction, and pure indirect effects. Bootstrap resampling (1000 iterations) was used to estimate confidence intervals.

In the multicollinearity test (Additional file: Table S7), the results revealed that the variance inflation factor (VIF) for every covariate was below 5, suggesting the absence of multicollinearity among the covariates [43].

Sensitivity analyses were performed on the data before multiple imputation, excluding participants with missing values, to confirm the reliability of the outcomes (Additional file: Table S8, Table S9). Furthermore, to address potential selection bias, we conducted a comparative analysis between participants included in the final cohort (*n* = 7,976) and those excluded (*n* = 9,729). Standardized mean differences (SMDs) were calculated for all baseline characteristics to assess the balance between the two groups. An SMD threshold of < 0.1 was predefined as indicative of negligible imbalance, following established recommendations [44, 45].

Statistical analyses were conducted using R software (version 4.4.2) and SPSS software (version 29.0.2.0). Two-sided P value α < 0.05 was considered statistically significant.

## Results

### Baseline characteristics of participants

A total of 7976 participants in CHARLS were enrolled (Table 1). The mean (SD) age was 58.68 (9.60) years, including 4263 (53.45%) females. The mean (SD) TyG-WHtR value was 4.68 (0.76). The mean (SD) MAP value was 93.58 (14.17) mmHg. During a maximum follow-up of 7.0 years, 1326 (16.62%) people developed CVD. Median follow-up duration was 7.0 years due to 6788 participants (85.11%) completing the full 7-year follow-up.

Demographic and clinical characteristics of the included cohort (*n* = 7,976) and excluded participants (*n* = 9,729) are summarized in Table S10. All variables exhibited SMDs below 0.1, indicating minimal imbalance between the groups. This suggests that the exclusion process did not systematically bias the analyzed population with respect to measured covariates.

**Table 1:** Baseline characteristics of 7976 participants

Characteristics				
Participants, No			7976	
Age, years, mean (SD)			58.68 (9.60)	
Gender, Female, n (%)			4263 (53.45)	
SBP, mmHg, mean(SD)			129.82 (21.35)	
DBP, mmHg, mean (SD)			75.46 (12.08)	
MAP, mmHg, mean (SD)			93.58 (14.17)	
Waist, cm, mean (SD)			84.82 (9.80)	
BMI, kg/m^2^, mean (SD)			23.29 (3.52)	
WHtR, mean (SD)			0.54 (0.06)	
HGB, g/dL, mean (SD)			14.33 (2.23)	
PLT, 10^9^/L, mean (SD)			211.71 (73.17)	
BUN, mg/dL, mean (SD)			15.72 (4.57)	
Scr, mg/dL, mean (SD)			0.78 (0.24)	
UA, mg/dL, mean (SD)			4.43 (1.24)	
FPG, mg/dL, mean (SD)			109.67 (36.68)	
HBA1C, %, mean (SD)			5.25 (0.80)	
TC, mg/dL, mean (SD)			193.23 (38.83)	
TG, mg/dL, mean (SD)			131.96 (109.87)	
HDL-c, mg/dL, mean (SD)			51.52 (15.30)	
LDL-c, mg/dL, mean (SD)			115.95 (35.00)	
eGFR, ml/minute/1.73m^2^, mean (SD)			109.22 (29.17)	
TyG.mean (SD)			8.67 (0.67)	
TyG-WHtR, mean (SD)			4.68 (0.76)	
Hypertension, n (%)			1323 (16.59)	
Diabetes, n (%)			370 (4.64)	
Cancer, n (%)			77 (0.97)	
Lung Diseases, n (%)			730 (9.15)	
Liver Diseases, n (%)			289 (3.62)	
CVD, n (%)			1326 (16.62)	
Marriage, married, n (%)			7062 (88.54)	
Depression, n (%)			3743 (46.93)	
Sleep problems, n (%)			3970 (49.77)	
Educational level, n (%)				
No completion of primary school			3825 (47.96)	
Sishu/home school/elementary school			1800 (22.57)	
Middle school			1584 (19.86)	
High school and above			767 (9.62)	
Smoking status, n (%)				
Never			4850 (60.81)	
Quit			647 (8.11)	
Still			2479 (31.08)	
Drinking status, n (%)				
Never			4840 (60.68)	
Quit			699 (8.76)	
Still			2437 (30.55)	

### The relationship between the TyG-WHtR, MAP and CVD

**Fig. 2:**
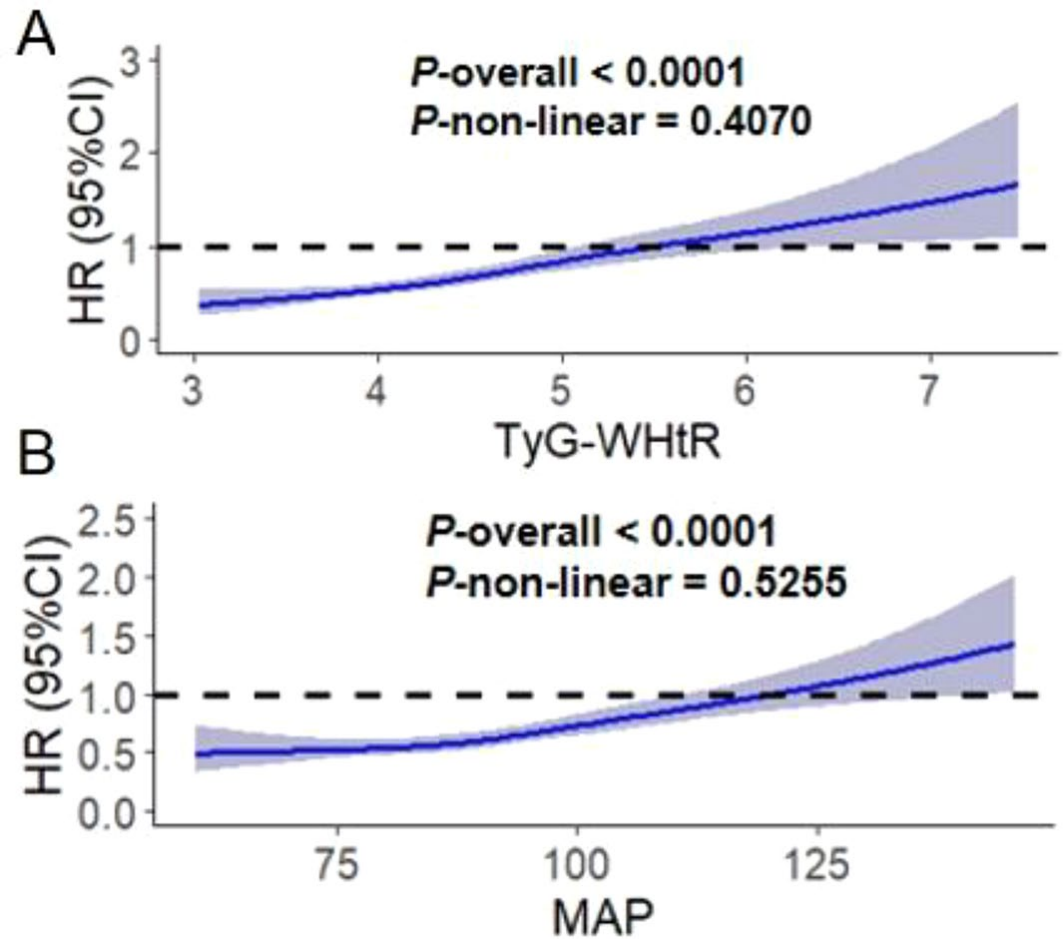
The RCS analysis between the TyG-WHtR, age and CVD risk. The model was adjusted for Age, Gender, HGB, PLT, BUN, Scr, UA, TC, HDL-c, LDL-c, Diabetes, Cancer, Lung disease, Liver disease, Education level, Marital status, Depression, Sleep problems, Smoking statues, Drinking statues

Figure 2 showed that restricted cubic spline models were employed to evaluate potential nonlinear relationships between TyG-WHtR, MAP, and CVD risk. For TyG-WHtR, the overall association with CVD was statistically significant (P-overall < 0.0001), with no evidence of nonlinearity (P-non-linear = 0.4070; Fig. 2A). Similarly, MAP demonstrated a strong linear association with CVD (P-overall < 0.0001; P-non-linear = 0.5255; Fig. 2B). These results support the use of linear models to characterize the dose-response relationships between TyG-WHtR, MAP, and CVD risk.

**Fig. 3:**
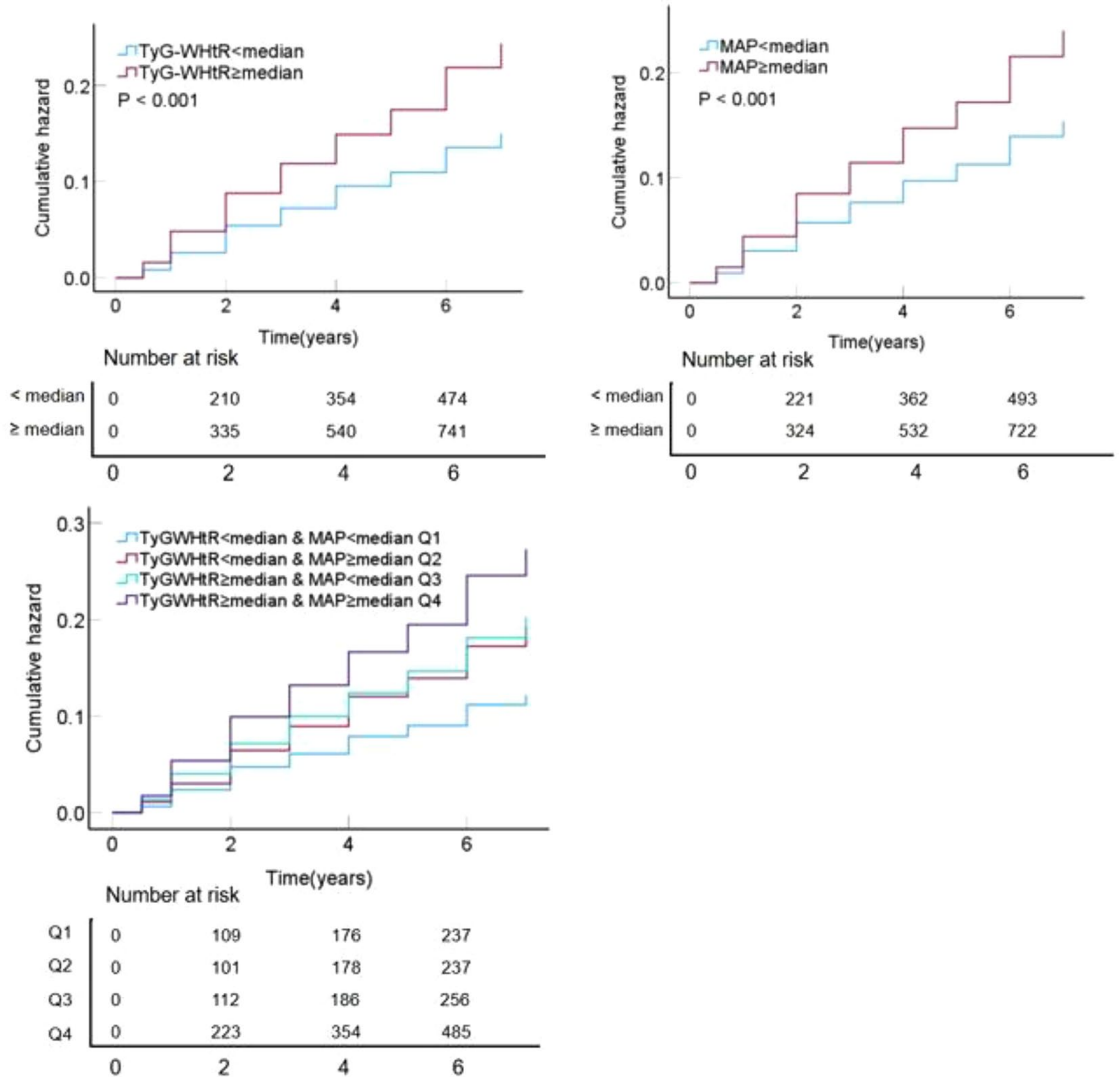
KM plot of CVD based on TyG-WHtR index and MAP

Figure 3 showed the cumulative incidence rates of CVD based on the TyG-WHtR index and MAP according to the KM plot. Participants with both high TyG-WHtR and high MAP exhibited the highest cumulative incidence of CVD events (log-rank *P* < 0.001). Post-hoc pairwise comparisons confirmed significant differences between this group and all other combinations (Table S11, Bonferroni-corrected threshold = 0.0083). Additionally, Supplementary Figure S1 illustrated the cumulative incidence rates of CVD based on the quartiles of the TyG-WHtR index and MAP, yielding consistent results.

**Fig. 4:**
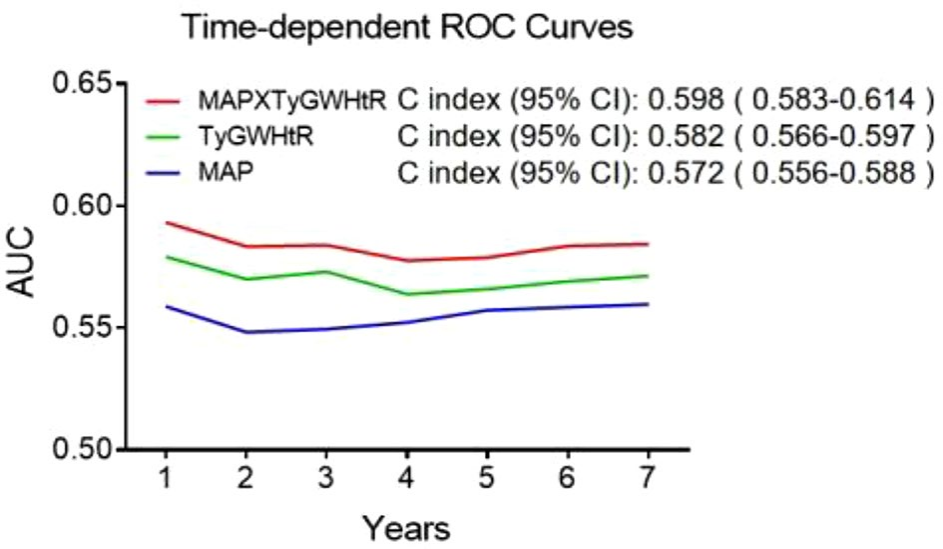
Time-Dependent ROC curves for CVD Prediction

Figure 4 presented the predictive capacity of the TyG-WHtR index, MAP, and MAP**×**TyG-WHtR for CVD, as assessed by ROC. Time-dependent ROC analysis demonstrated that the combined MAP**×**TyG-WHtR index produced a C-index of 0.598 (95% CI 0.583–0.614), which was higher than TyG-WHtR alone (C-index: 0.582, 95% CI 0.566–0.597) and MAP alone (C-index: 0.572, 95% CI 0.556–0.588).

Table 2 showed the IDI and NRI to evaluate the incremental utility of combining TyG-WHtR with MAP. Compared to MAP alone, the addition of TyG-WHtR resulted in an IDI of 0.008 (95% CI 0.004–0.013, *P* < 0.001) and an NRI of 0.100 (95% CI 0.056–0.141, *P* < 0.001). Similarly, compared to TyG-WHtR alone, the addition of MAP improved prediction with an IDI of 0.006 (95% CI 0.002–0.011, *P* < 0.001) and an NRI of 0.058 (95% CI 0.005–0.116, *P* = 0.03).

**Table 2:** Improvement in CVD prediction with combined MAP×TyG-WHtR index

	IDI (95%CI)	*P*-value	NRI (95%CI)	*P*-value
MAP**×**TyGWHtR vs. MAP	0.008 (0.004–0.013)	< 0.001	0.100 (0.056–0.141)	< 0.001
MAP**×**TyGWHtR vs. TyGWHtR	0.006 (0.002–0.011)	< 0.001	0.058 (0.005–0.116)	0.030

MAP×TyGWHtR: the combination of TyG-WHtR index and MAP; IDI: integrated discrimination improvement; NRI: net reclassification improvement

Table 3 showed the association of TyG-WHtR index and MAP with CVD incidence according to three Cox proportional hazards models. In the fully adjusted Model III, compared with participants with a lower TyG-WHtR index (≤ median level) and MAP (≤ median level), those with a higher TyG-WHtR index (≥ median level) and MAP (≥ median level) had the highest risk of CVD (HR: 1.961; 95%CI 1.660–2.317).

Additionally, in Table S4, TyG-WHtR index was categorized into four groups according to quartiles. In the fully adjusted Model III, each 1-unit increase in TyG-WHtR was associated with a 44.6% higher CVD risk (HR = 1.446, 95% CI 1.314–1.591). Per 1-SD increment (SD = 0.76 unit), the HR was 1.312 (95% CI 1.224–1.453), corresponding to a 31.2% elevated risk. Then, compared to the first quartile (Q1), the HR for Q2, Q3, and Q4 were 1.235 (95%CI 1.034–1.475), 1.565 (95%CI 1.310–1.870), and 1.883 (95%CI 1.544–2.296), respectively. ‌Similarly‌, in Table S5, MAP was grouped into four according to quartiles. In the fully adjusted Model III, each 1-mmHg increase in MAP was associated with a 1.5% higher CVD risk (HR = 1.015, 95% CI 1.011–1.019). When scaled per 1-SD increment (SD = 14.17 mmHg), the HR increased to 1.246 (95% CI 1.174–1.323), corresponding to a 24.6% elevated risk. Compared to the first quartile (Q1), the HR for Q2, Q3, and Q4 were 1.089 (95%CI 0.919–1.291), 1.364 (95%CI 1.159–1.605), and 1.635 (95%CI 1.393–1.919), respectively. Then, the combination of TyG-WHtR index and MAP was grouped into sixteen according to the quartiles of TyG-WHtR index and MAP, as showed in Table S6, In the Model III, compared with participants with a lower TyG-WHtR index (Q1) and MAP (Q1), those with a higher TyG-WHtR index (Q4) and MAP (Q4) exhibited the greatest CVD risk (HR: 2.248; 95%CI 1.674–3.018).

**Table 3:** Associations of the TyG-WHtR index and MAP with the risk of CVD

	Model I	*P*-value	Model II	*P*-value	Model III	*P*-value
	HR (95% CI)		HR (95% CI)		HR (95% CI)	
TyG-WHtR < median & MAP < median	Ref		Ref		Ref	
TyG-WHtR < median & MAP ≥ median	1.561 (1.313–1.854)	< 0.001	1.465 (1.231–1.743)	< 0.001	1.501 (1.261–1.786)	< 0.001
TyG-WHtR ≥ median & MAP < median	1.642 (1.387–1.945)	< 0.001	1.535 (1.281–1.839)	< 0.001	1.523 (1.270–1.827)	< 0.001
TyG-WHtR ≥ median & MAP ≥ median	2.200 (1.895–2.554)	< 0.001	1.927 (1.634–2.273)	< 0.001	1.961 (1.660–2.317)	< 0.001

Model I: Crude model; Model II: Adjusted for Age, Gender, HGB, PLT, BUN, Scr, UA, TC, HDL-c, LDL-c; Model III: Adjusted for Age, Gender, HGB, PLT, BUN, Scr, UA, TC, HDL-c, LDL-c, Diabetes, Cancer, Lung disease, Liver disease, Education level, Marital status, Depression, Sleep problems, Smoking statues, Drinking statues

**Fig. 5:**
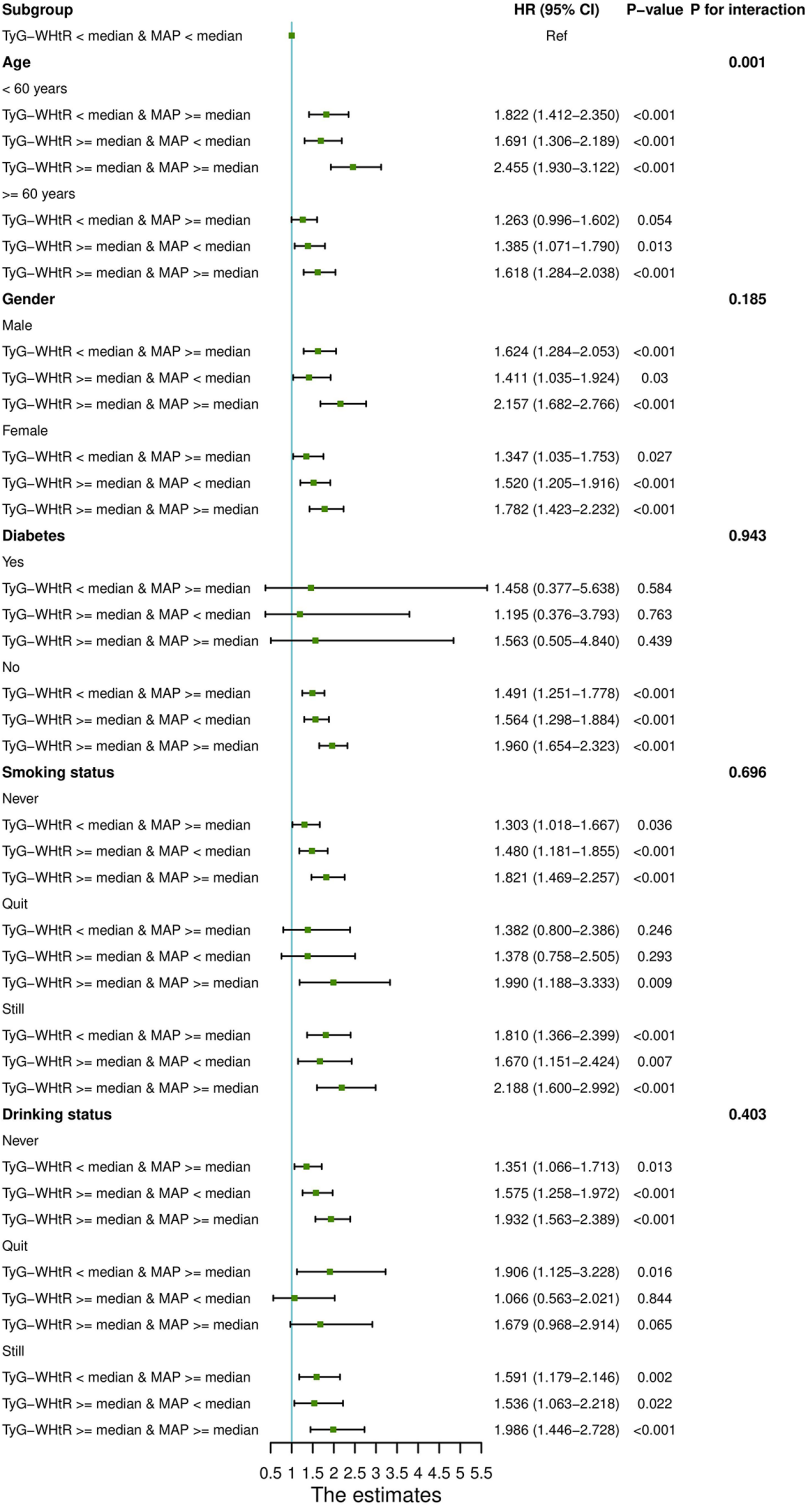
Subgroup analyses of the association of the TyG-WHtR and MAP with the risk of CVD. Age, Gender, HGB, PLT, BUN, Scr, UA, TC, HDL-c, LDL-c, Diabetes, Education level, Marital status, Depression, Sleep problems, Smoking statues, Drinking statues were adjusted, if not stratifed

Figure 5 showed the subgroup and interaction analyses across different age groups, genders, diabetes statuses, smoking and drinking statuses. The results showed interaction effects only among different age groups (*P* for interaction = 0.001), with no interactions observed in other subgroups (*P* for interaction > 0.05). The corresponding numerical results were showed in Table S12.

**Fig. 6:**
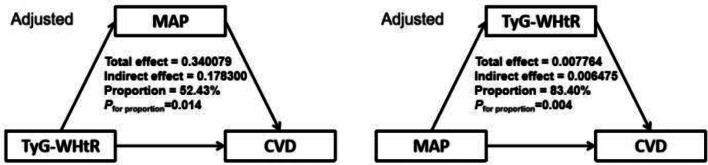
Mediation effects of TyG-WHtR index and MAP in the incidence of CVD. Adjusted for Age, Gender, HGB, PLT, BUN, Scr, UA, TC, HDL-c, LDL-c, Diabetes, Cancer, Lung disease, Liver disease, Education level, Marital status, Depression, Sleep problems, Smoking statues, Drinking statues

Figure 6 showed the potential mediating effects of increased TyG-WHtR index and MAP in the incidence of CVD. In exploratory four-way decomposition analyses, MAP mediated 52.43% of the association between TyG-WHtR and CVD (*P* = 0.014), while TyG-WHtR mediated 83.40% of the MAP-CVD association (*P* = 0.004), with no significant interaction (*P* = 0.106, Table S13, Table S14). These bidirectional mediation proportions should be interpreted as hypothesis-generating, reflecting intertwined metabolic and hemodynamic dysregulation rather than strict causal sequences.

## Discussion

Among 7976 participants from CHARLS followed up to 7.0 years, a higher TyG-WHtR index and a higher MAP were signifcantly associated with a higher risk of CVD. The highest risk of CVD incidence was observed among those with a higher TyG-WHtR index and a higher MAP. Furthermore, this study indicated that the elevated TyG-WHtR index partially mediated the relationship between MAP and CVD, while elevated MAP also partially mediated the association between TyG-WHtR index and CVD. Importantly, we found that the combination of TyG-WHtR index and MAP enhanced the predictive capability for CVD. Based on the literature search, this study is the first to explore the complex relationships and mediating effects of TyG-WHtR index and MAP in CVD. Considering the complex relationship between blood pressure, IR, and CVD, it is necessary to investigate the complex relationships of TyG-WHtR index and MAP in CVD.

Blood pressure played a crucial role in the occurrence and development of CVD, which was a well-established risk factor supported by extensive evidence [5]. As blood pressure increased, the mechanical stress on the arterial wall was elevated, leading to thickening, stiffening, and decreased elasticity of the vessel wall [46], ultimately resulting in vascular remodeling and fibrosis [47]. Endothelial cells were essential for maintaining vascular homeostasis, and elevated blood pressure could damage endothelial cells, with endothelial dysfunction contributing to the development of atherosclerosis and plaque formation [48]. Increased blood pressure was often associated with chronic inflammation and immune cell activation [49], and this immune activation might lead to vascular inflammation and the formation of atherosclerotic plaques. Furthermore, elevated blood pressure was typically accompanied by enhanced sympathetic nervous system activity [50], which could induce vasoconstriction and further increase blood pressure, creating a vicious cycle. In this study, MAP was used to represent the participants’ blood pressure levels, and the results showed a positive correlation between MAP levels and the risk of CVD, which was consistent with findings from related studies. Higher MAP levels were significantly associated with an increased risk of target organ damage [10–12].

Elevated blood pressure could lead to IR, another risk factor for CVD, through mechanisms involving chronic inflammation [51] and sympathetic nervous system activation [52]. Studies had shown that reducing peripheral catecholamine levels by knocking out tyrosine hydroxylase in mouse, could significantly alleviate the IR induced by sympathetic nervous system activation [52]. Increased sympathetic tone triggered the activation of β-adrenergic receptors, which in turn activated serine/threonine kinases. This activation blunted insulin metabolic signaling, thereby contributing to the development of IR [53], suggesting that IR partially mediated the relationship between elevated blood pressure and CVD. This study revealed that the TyG-WHtR index, a representative indicator of IR, exhibited a significant 83.40% mediating effect in the relationship between MAP and CVD.

IR was another well-recognized risk factor for CVD, which had been extensively demonstrated in clinical studies. IR was significantly associated with the progression of coronary artery plaques [54], cardiac sudden death [55], recurrence of atrial fibrillation [56], and the risk of stroke [57]. In this study, the TyG-WHtR index, which combined the TyG index and the obesity indicator WHtR, was considered an effective representative indicator of IR and was positively correlated with the risk of CVD.

IR contributed to an elevated blood pressure through several interconnected molecular mechanisms. In the endothelium, IR impaired the PI3K-Akt-eNOS pathway, reducing nitric oxide (NO) production, which was crucial for maintaining vascular relaxation [58]. The activation of the epithelial sodium channel by insulin and aldosterone through serum and glucocorticoid kinase 1 (SGK-1) led to increased Na + influx, cytoskeletal remodeling, and reduced NO bioavailability, contributing to vascular stiffness [59]. In the kidneys, insulin resistance promoted microvascular remodeling, inflammation, and oxidative stress, leading to glomerulosclerosis, albuminuria, and renal fibrosis [60]. Additionally, insulin signaling defects in podocytes and tubular epithelial cells disrupted glomerular filtration barrier integrity, leading to albuminuria and progressive renal dysfunction [61]. These mechanisms collectively resulted in increased blood pressure, as endothelial dysfunction and vascular stiffness impair blood flow and increase vascular resistance, while kidney damage disrupts the balance of fluid and electrolytes, further exacerbating hypertension. This suggested that elevated blood pressure played a partial mediating role between IR and CVD. This study revealed that MAP had a significant 52.43% mediating effect between TyG-WHtR index and CVD.

The bidirectional TyG-WHtR-MAP association reflects a metabolic-vascular axis wherein insulin resistance and hypertension mutually reinforce CVD risk. Mechanistically, angiotensin II and endothelin-1 directly inhibited insulin receptor substrate-1 phosphorylation, promoting systemic insulin resistance [62, 63], while hyperinsulinemia stimulated vascular smooth muscle cell proliferation and endothelin-1 secretion, further increasing vascular resistance [63, 64]. This interplay was further validated by therapies targeting both pathways [65], which reduced CVD events beyond glycemic or blood pressure alone.

We found that the risk of CVD in the group with a higher TyG-WHtR index and a higher MAP was 1.961 times than that in the group with a lower TyG-WHtR index and a lower MAP among 7976 participants during a follow-up period of 7.0 years. These findings helped elucidate the predictive value of the combined analysis of the TyG-WHtR index and MAP for CVD, thereby enabling more accurate identification of individuals at high risk of CVD. Our research could provide in-depth insights into the complex relationship between IR and blood pressure in CVD.

Although the absolute C-index of the combined MAP×TyG-WHtR index indicated modest discriminatory capacity, the statistically significant improvements in IDI and NRI suggested that integrating TyG-WHtR with MAP provided incremental predictive value for CVD risk stratification.

Subgroup analyses revealed a statistically significant interactions of the combined effects of TyG-WHtR index and MAP between different age groups, suggesting that managing the TyG-WHtR index and MAP in participants < 60 years could considerably lower the incidence of CVD compared to those ≥ 60 years.

The present study offers several clear advantages: First, it is a prospective, large-scale cohort study that, for the first time, evaluated the relationship between the TyG-WHtR index, MAP, and CVD, and investigated the mediating role of these factors in CVD. Second, we explored the nature of the association between the TyG-WHtR index, MAP, and CVD risk, revealing a linear relationship. Third, the subgroup analysis revealed that a heightened focus on the combined effects of the TyG-WHtR index and MAP among participants under 60 years of age could substantially reduce the incidence of CVD in this demographic group. Fourth, we also examined the predictive ability of the TyG-WHtR index and MAP for CVD using Time-dependent ROC analysis. Furthermore, sensitivity analyses were performed to assess the stability and reliability of our results.

The present study also has several limitations. First, due to the observational nature of the research, we were unable to establish a causal relationship between the TyG-WHtR index, MAP and CVD. The cross-sectional measurement of TyG-WHtR and MAP precludes definitive conclusions about causal directionality in mediation pathways. Although four-way decomposition accounts for interaction and confounding, longitudinal or interventional studies are needed to validate these mechanisms. Second, despite adjusting for potential cardiac risk factors and excluding MAP and TyG-WHtR components to avoid collinearity, we cannot completely rule out the presence of residual or unmeasured confounding factors (such as medication) inherent to the observational study design. Third, CVD diagnosis was based on self-reports from CHARLS participants, which may introduce some deviation from actual incidence rates. However, it was worth noting that previous research had indicated that self-reports were largely consistent with medical records, and misreporting was not systematically biased, suggesting that any potential misclassification bias was minimal [66, 67]. Fourth, as all participants were from the Chinese population, the generalizability of these findings to other countries may be limited, and further studies are required to validate these results in different populations and countries. Fifth, the blood pressure data in this study were based on measurements taken from the left arm, which may not fully capture the subtle differences in blood pressure in the right arm. Future studies could further validate the universality of the results by measuring both arms.

## Conclusions

This cohort study demonstrated that the combination of a higher TyG-WHtR index and a higher MAP was associated with the highest risk of CVD. The combination of TyG-WHtR and MAP provided incremental predictive utility for CVD over individual biomarkers, evidenced by significant improvements in IDI and NRI. Although the absolute predictive performance remains moderate. The MAP could partially mediate the association between TyG-WHtR index and CVD, as well as TyG-WHtR index could also partially mediate the association between MAP and CVD. These findings recommend the combined assessment of the TyG-WHtR index and MAP to further stratify the risk of CVD. More attention is needed to the complex relationship between blood pressure, IR, and CVD.

## Electronic supplementary material

Below is the link to the electronic supplementary material.

Supplementary Material 1

## Data Availability

Online repositories contain the datasets used in this study. The names of the repositories and accession numbers can be found at http://charls.pku.edu.cn/en.

## Abbreviations

CVD: Cardiovascular diseases
MAP: Mean arterial pressure
TyG-WHtR: Triglyceride glucose-waist height ratio
IR: Insulin resistance
TyG: Triglyceride glucose index
WHtR: Waist height ratio
BMI: Body mass index
SBP: Systolic blood pressure
DBP: Diastolic blood pressure
TG: Tiglyceride
TC: Total cholesterol
HDL-c: High-density lipoprotein cholesterol
LDL-c: Low-density lipoprotein cholesterol
HGB: Hemoglobin
PLT: Platelets
FPG: Fasting plasma glucose
HbA1c: Glycated hemoglobin a1c
BUN: Blood urea nitrogen
Scr: Serum creatinine
UA: Uric acid
RCS: Restricted cubic spline
ROC: Receiver operating characteristic
AUC: Area under the curve
CHARLS: China health and retirement longitudinal study
eGFR: Estimated glomerular filtration rate
C-MDRD: Chinese modification of diet in renal disease
CESD10: 10-item short form of the center for epidemiologic studies depression scale
KM: Kaplan-Meier plot
SD: Standard deviations
ANOVA: Analysis of variance
VIF: Variance inflation factor
HR: Hazard ratio
CI: Confidence interval
SMDs: Standardized mean differences
CDC: Chinese center for disease control and prevention
IDI: Integrated discrimination improvement
NRI: Net reclassification improvement
