# Correction: Insights into the complex relationship between triglyceride glucose-waist height ratio index, mean arterial pressure, and cardiovascular disease: a nationwide prospective cohort study

**DOI:** 10.1186/s12933-025-02728-2

**Published:** 2025-05-15

**Authors:** Jie Xu, Dihui Cai, Yuheng Jiao, Yingying Liao, Yinyin Shen, Yunli Shen, Wei Han

**Affiliations:** 1 Department of Cardiology, Shanghai East Hospital, School of Medicine, Tongji University, Shanghai, China; 2 State Key Laboratory of Cardiology and Medical Innovation Center, Shanghai East Hospital, School of Medicine, Tongji University, Shanghai, China

**Correction to: Cardiovascular Diabetology (2025) 24:93** 10.1186/s12933-025-02657-0

In the original publication of this article [1], Figs. 1 and 2 were inadvertently duplicated and Figs. 3, 4, 5, and 6 contained incorrect images. For completeness and transparency, the correct version of Figs. 1–6 are displayed below:

**Fig. 1:**
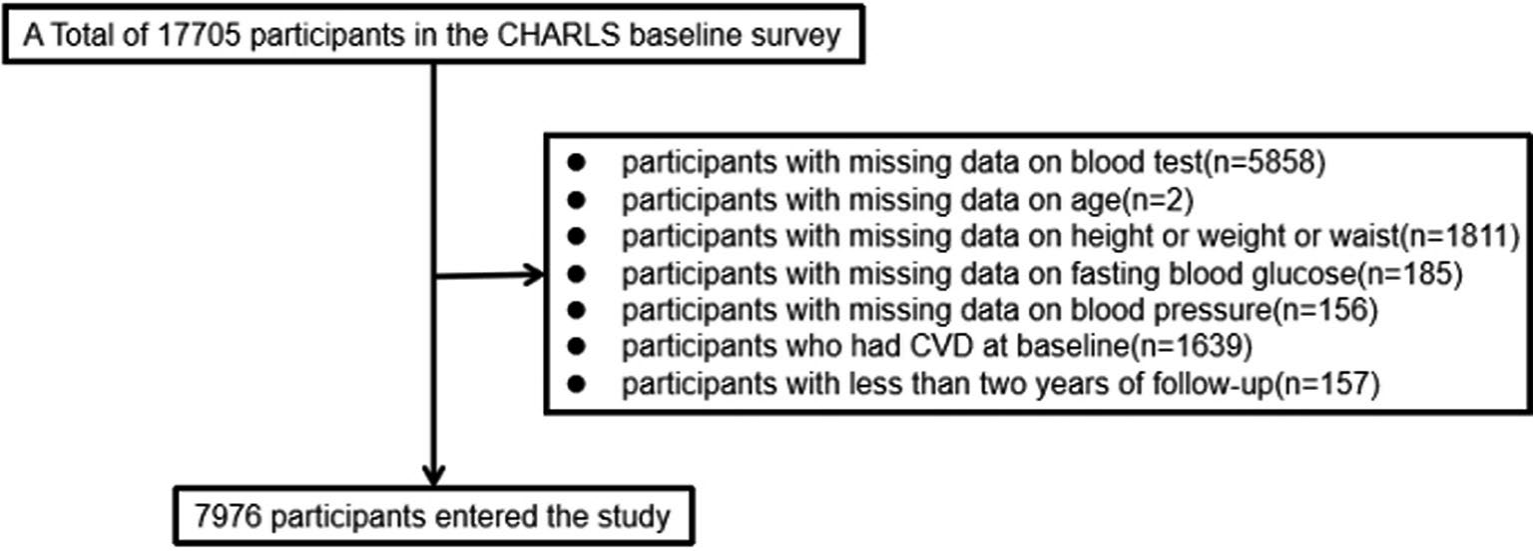
Flowchart of the study population

**Fig. 2:**
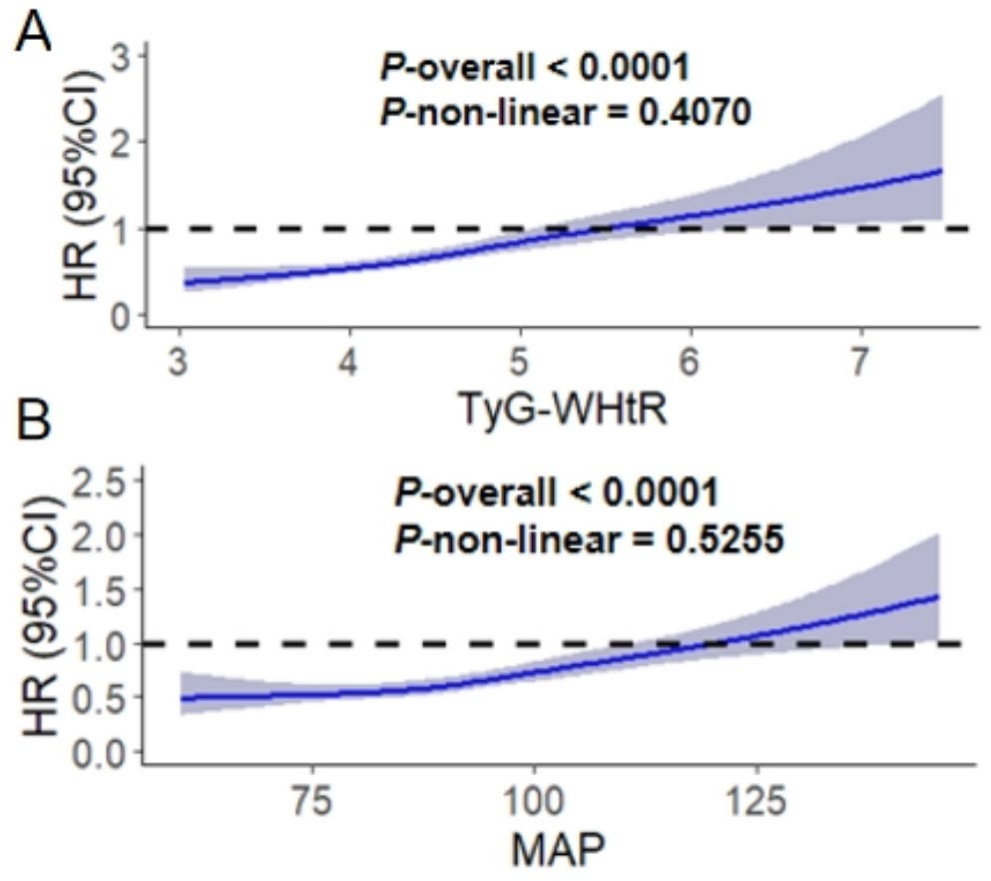
The RCS analysis between the TyG-WHtR, age and CVD risk. The model was adjusted for Age, Gender, HGB, PLT, BUN, Scr, UA, TC, HDL-c, LDL-c, Diabetes, Cancer, Lung disease, Liver disease, Education level, Marital status, Depression, Sleep problems, Smoking statues, Drinking statues

**Fig. 3:**
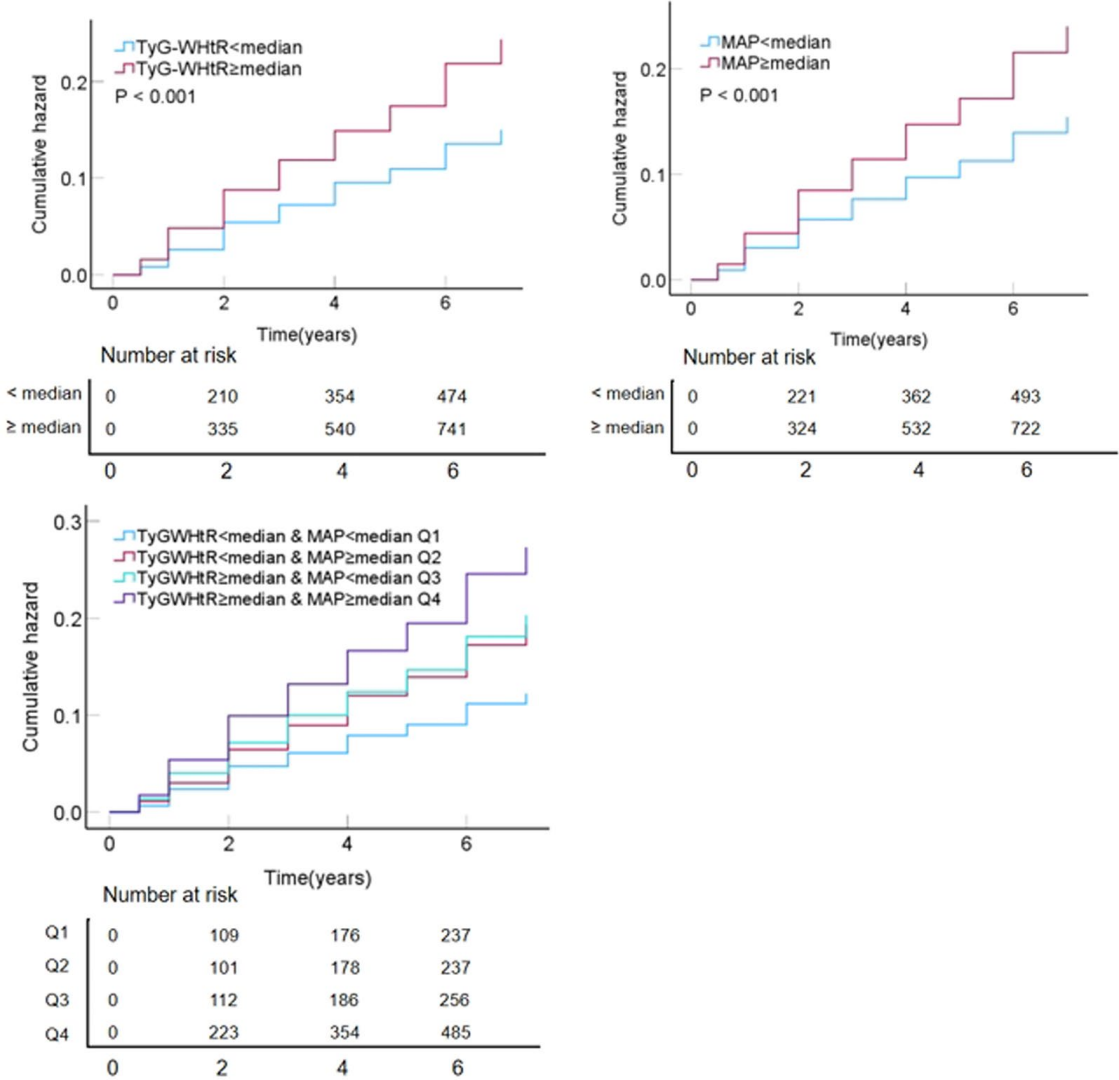
KM plot of CVD based on TyG-WHtR index and MAP

**Fig. 4:**
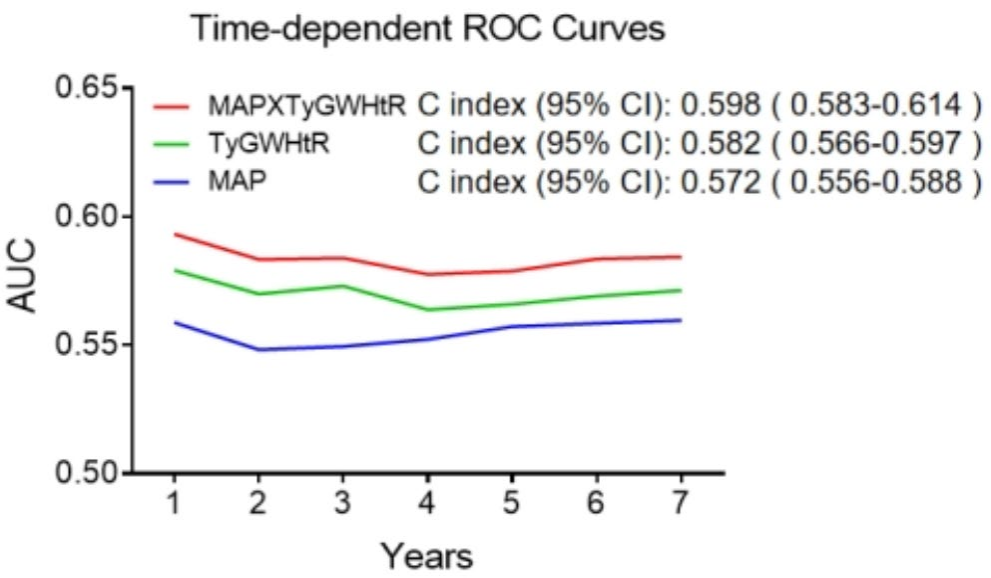
Time-Dependent ROC curves for CVD Prediction

**Fig. 5:**
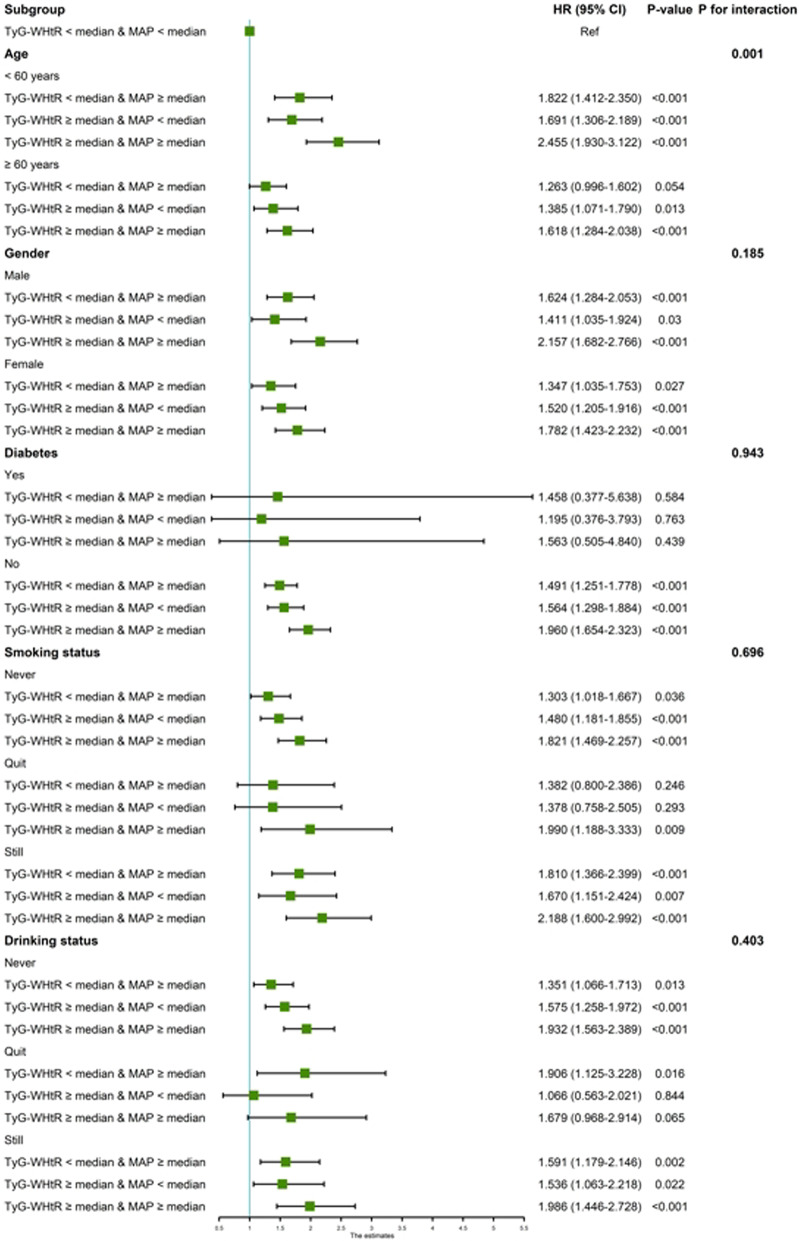
Subgroup analyses of the association of the TyG-WHtR and MAP with the risk of CVD. Age, Gender, HGB, PLT, BUN, Scr, UA, TC, HDL-c, LDL-c, Diabetes, Education level, Marital status, Depression, Sleep problems, Smoking statues, Drinking statues were adjusted, if not stratifed

**Fig. 6:**
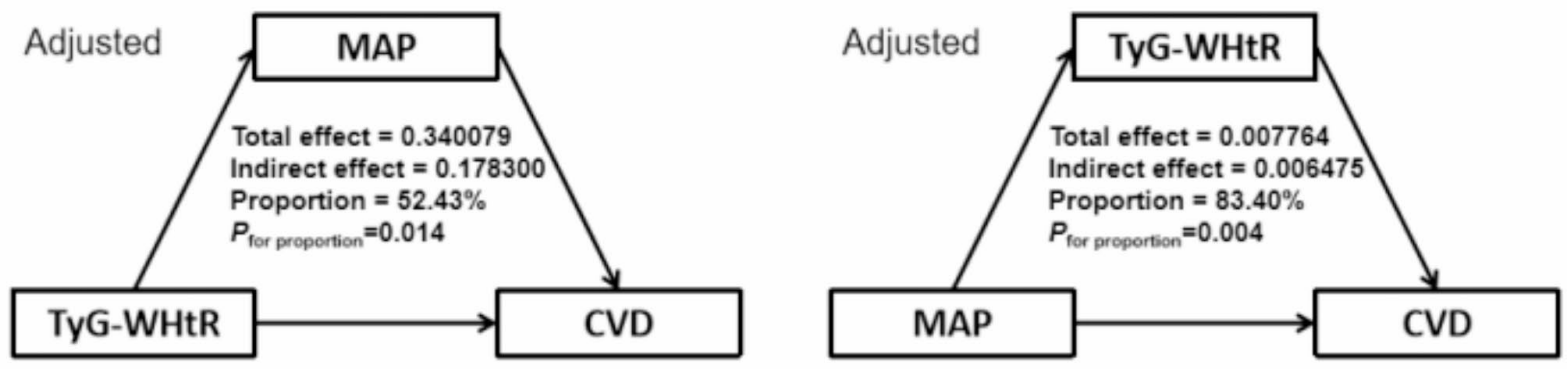
Mediation effects of TyG-WHtR index and MAP in the incidence of CVD. Adjusted for Age, Gender, HGB, PLT, BUN, Scr, UA, TC, HDL-c, LDL-c, Diabetes, Cancer, Lung disease, Liver disease, Education level, Marital status, Depression, Sleep problems, Smoking statues, Drinking statues

The original article has been corrected.
