# Supplementary material for: Insights into the complex relationship between triglyceride glucose-waist height ratio index, mean arterial pressure, and cardiovascular disease: a nationwide prospective cohort study
Source: Cardiovasc Diabetol. 2025 Feb 28;24:93. doi: 10.1186/s12933-025-02657-0 (PMC11871683; doi:10.1186/s12933-025-02657-0)
Supplement: Supplementary file 1 — Supplementary Material 1 [file 12933_2025_2657_MOESM1_ESM.docx]

**Table S1. Distribution of variables with missing data**

| Variables | Number of Missing | Missing proportion |
| --- | --- | --- |
| Depression | 236 | 2.96% |
| HGB | 149 | 1.87% |
| PLT | 147 | 1.84% |
| Sleep problems | 113 | 1.42% |
| Liver Disease | 68 | 0.85% |
| HBA1C | 62 | 0.78% |
| Cancer | 45 | 0.56% |
| Lung Diseases | 39 | 0.49% |
| LDL-c | 14 | 0.17% |
| Smoking statues | 8 | 0.10% |
| Drinking statues | 7 | 0.09% |
| Scr | 4 | 0.05% |
| BUN | 2 | 0.02% |
| HDL-c | 1 | 0.01% |

**Table S2. Baseline characteristics according to the TyG-WHtR index quartiles**

| Characteristics | TyG-WHtR Q1 ～4.11 | TyG-WHtR Q2 4.11～4.60 | TyG-WHtR Q3 4.60～5.15 | TyG-WHtR Q4 5.15～ | P-value |
| --- | --- | --- | --- | --- | --- |
|  |  |  |  |  |  |
| Age,years,mean(SD) | 58.75(9.87) | 58.03(9.51) | 58.61(9.49) | 59.32(9.48) | <0.001 |
| Gender,Female,n(%) | 694(34.8) | 993(49.8) | 1153(57.9) | 1423(71.4) | <0.001 |
| SBP,mmHg,mean(SD) | 124.34(20.43) | 126.52(19.95) | 131.24(20.79) | 137.17(21.91) | <0.001 |
| DBP,mmHg,mean(SD) | 72.34(11.82) | 73.99(11.79) | 76.29(11.70) | 79.21(11.89) | <0.001 |
| MAP,mmHg,mean(SD) | 89.68(13.88) | 91.50(13.57) | 94.60(13.69) | 98.53(13.91) | <0.001 |
| Waist,cm,mean(SD) | 74.90(5.67) | 81.39(5.57) | 87.70(6.09) | 95.30(7.49) | <0.001 |
| BMI,kg/m2，mean(SD) | 20.15(2.11) | 22.21(2.25) | 24.06(2.55) | 26.74(3.18) | <0.001 |
| WHtR,mean(SD) | 0.47(0.03) | 0.51(0.03) | 0.56(0.03) | 0.61(0.04) | <0.001 |
| HGB,g/dL,mean(SD) | 14.19(2.21) | 14.32(2.26) | 14.30(2.15) | 14.50(2.30) | <0.001 |
| PLT,109/L,mean(SD) | 207.49(73.40) | 211.78(71.53) | 211.66(73.08) | 215.90(74.45) | 0.004 |
| BUN,mg/dL,mean(SD) | 16.24(4.86) | 15.72(4.68) | 15.59(4.39) | 15.32(4.29) | <0.001 |
| Scr,mg/dL,mean(SD) | 0.79(0.21) | 0.78(0.22) | 0.77(0.30) | 0.76(0.19) | <0.001 |
| UA,mg/dL,mean(SD) | 4.32(1.17) | 4.34(1.23) | 4.44(1.27) | 4.63(1.28) | <0.001 |
| Glucose,mg/dL,mean(SD) | 98.28(17.71) | 104.04(24.93) | 108.47(28.61) | 127.90(55.99) | <0.001 |
| HBA1C,%,mean(SD) | 5.06(0.50) | 5.15(0.62) | 5.23(0.68) | 5.57(1.15) | <0.001 |
| TC,mg/dL,mean(SD) | 181.30(34.66) | 188.85(35.19) | 196.39(36.59) | 206.40(43.71) | 0.029 |
| TG,mg/dL,mean(SD) | 76.09(31.77) | 103.02(47.36) | 131.03(63.84) | 217.74(172.20) | <0.001 |
| HDL-c,mg/dL,mean(SD) | 59.71(15.57) | 54.19(14.52) | 49.42(13.28) | 42.75(12.29) | <0.001 |
| LDL-c,mg/dL,mean(SD) | 108.32(30.77) | 115.61(31.37) | 121.47(34.11) | 118.42(41.37) | <0.001 |
| eGFR,ml/minute/1.73m2,mean(SD) | 110.60(28.38) | 109.52(27.23) | 108.56(27.60) | 108.20(33.06) | 0.044 |
| TyG.mean(SD) | 8.14(0.42) | 8.48(0.43) | 8.75(0.48) | 9.30(0.69) | <0.001 |
| TyG-WHtR,mean(SD) | 3.79(0.23) | 4.36(0.14) | 4.87(0.16) | 5.70(0.46) | <0.001 |
| Hypertension,n(%) | 154(7.7) | 216(10.8) | 371(18.6) | 582(29.2) | <0.001 |
| Diabetes,n(%) | 10(0.5) | 37(1.9) | 65(3.3) | 258(12.9) | <0.001 |
| Cancer,n(%) | 24(1.2) | 15(0.8) | 9(0.5) | 29(1.5) | 0.006 |
| Lung Diseases,n(%) | 206(10.3) | 198(9.9) | 157(7.9) | 169(8.5) | 0.021 |
| Liver Disease,n(%) | 66(3.3) | 76(3.8) | 71(3.6) | 76(3.8) | 0.803 |
| CVD,n(%) | 230(11.5) | 286(14.3) | 363(18.2) | 447(22.4) | <0.001 |
| Marriage,married,n(%) | 1765(88.5) | 1780(89.3) | 1778(89.2) | 1739(87.2) | 0.144 |
| Depression,n(%) | 947(47.5) | 935(46.9) | 934(46.9) | 927(46.5) | 0.942 |
| Sleep problems,n(%) | 992(49.7) | 1004(50.4) | 964(48.4) | 1010(50.7) | 0.483 |
| Educational level,n(%) |  |  |  |  | 0.002 |
| No completion of primary school | 914(45.8) | 925(46.4) | 947(47.5) | 1039(52.1) |  |
| Sishu/home school/elementary school | 469(23.5) | 474(23.8) | 447(22.4) | 410(20.6) |  |
| Middle school | 419(21.0) | 379(19.0) | 404(20.3) | 382(19.2) |  |
| High school and above | 193(9.7) | 216(10.8) | 195(9.8) | 163(8.2) |  |
| Smoking status,n(%) |  |  |  |  | <0.001 |
| Never | 907(45.5) | 1174(58.9) | 1278(64.1) | 1491(74.8) |  |
| Quit | 160(8.0) | 156(7.8) | 186(9.3) | 145(7.3) |  |
| Still | 928(46.5) | 664(33.3) | 529(26.5) | 358(18.0) |  |
| Drinking status,n(%) |  |  |  |  | <0.001 |
| Never | 1027(51.5) | 1184(59.4) | 1240(62.2) | 1389(69.7) |  |
| Quit | 184(9.2) | 167(8.4) | 192(9.6) | 156(7.8) |  |
| Still | 784(39.3) | 643(32.2) | 561(28.1) | 449(22.5) |  |

**Table S3. Baseline characteristics according to MAP quartiles**

| Characteristics | MAP Q1 ～83.33 | MAP Q2 83.44～92.11 | MAP Q3 92.22～101.89 | MAP Q4 102.00～ | *P*-value |
| --- | --- | --- | --- | --- | --- |
|  |  |  |  |  |  |
| Age,years,mean(SD) | 57.17(9.27) | 57.98(9.41) | 59.34(9.64) | 60.22(9.78) | <0.001 |
| Gender,Female,n(%) | 1130(56.6) | 1075(54.1) | 1006(50.4) | 1052(52.8) | 0.001 |
| SBP,mmHg,mean(SD) | 107.55(7.83) | 121.21(7.92) | 133.38(8.81) | 157.13(17.11) | <0.001 |
| DBP,mmHg,mean(SD) | 61.66(5.32) | 71.27(4.07) | 78.59(4.59) | 90.32(8.87) | <0.001 |
| MAP,mmHg,mean(SD) | 76.97(4.97) | 87.92(2.50) | 96.85(2.76) | 112.58(9.16) | <0.001 |
| Waist,cm,mean(SD) | 81.43(8.64) | 84.04(9.44) | 85.74(9.72) | 88.08(10.15) | <0.001 |
| BMI,kg/m^2^，mean(SD) | 22.22(3.12) | 22.98(3.31) | 23.59(3.52) | 24.37(3.75) | <0.001 |
| WHtR,mean(SD) | 0.52(0.06) | 0.53(0.06) | 0.54(0.06) | 0.56(0.07) | <0.001 |
| HGB,g/dL,mean(SD) | 13.88(2.10) | 14.30(2.21) | 14.49(2.23) | 14.66(2.31) | <0.001 |
| PLT,10^9^/L,mean(SD) | 209.20(74.61) | 209.88(70.92) | 212.08(72.66) | 215.67(74.30) | 0.024 |
| BUN,mg/dL,mean(SD) | 15.55(4.68) | 15.79(4.46) | 15.79(4.43) | 15.75(4.71) | 0.276 |
| Scr,mg/dL,mean(SD) | 0.76(0.24) | 0.77(0.18) | 0.78(0.30) | 0.79(0.21) | <0.001 |
| UA,mg/dL,mean(SD) | 4.20(1.12) | 4.37(1.22) | 4.51(1.27) | 4.64(1.31) | <0.001 |
| Glucose,mg/dL,mean(SD) | 104.35(29.01) | 108.50(34.56) | 113.09(41.97) | 112.73(39.17) | <0.001 |
| HBA1C,%,mean(SD) | 5.17(0.65) | 5.23(0.75) | 5.31(0.92) | 5.30(0.86) | <0.001 |
| TC,mg/dL,mean(SD) | 186.72(36.00) | 191.69(37.59) | 196.16(40.17) | 198.36(40.40) | <0.001 |
| TG,mg/dL,mean(SD) | 116.77(92.48) | 124.48(84.83) | 138.14(118.71) | 148.46(133.60) | <0.001 |
| HDL-c,mg/dL,mean(SD) | 53.14(15.20) | 51.70(14.88) | 50.98(15.64) | 50.27(15.32) | <0.001 |
| LDL-c,mg/dL,mean(SD) | 111.61(32.20) | 115.30(34.17) | 118.21(36.69) | 118.70(36.30) | <0.001 |
| eGFR,ml/minute/1.73m^2^,mean(SD) | 112.48(30.04) | 110.22(28.56) | 107.72(28.23) | 106.45(29.45) | <0.001 |
| TyG.mean(SD) | 8.52(0.61) | 8.63(0.63) | 8.73(0.69) | 8.79(0.71) | <0.001 |
| TyG-WHtR,mean(SD) | 4.43(0.66) | 4.61(0.71) | 4.75(0.75) | 4.93(0.80) | <0.001 |
| Hypertension,n(%) | 3(0.2) | 71(3.6) | 387(19.4) | 862(43.2) | <0.001 |
| Diabetes,n(%) | 56(2.8) | 84(4.2) | 116(5.8) | 114(5.7) | <0.001 |
| Cancer,n(%) | 24(1.2) | 21(1.1) | 19(1.0) | 13(0.7) | 0.335 |
| Lung Diseases,n(%) | 185(9.3) | 189(9.5) | 193(9.7) | 163(8.2) | 0.354 |
| Liver Disease,n(%) | 98(4.9) | 75(3.8) | 63(3.2) | 53(2.7) | 0.001 |
| CVD,n(%) | 256(12.8) | 282(14.2) | 360(18.0) | 428(21.5) | <0.001 |
| Marriage,married,n(%) | 1829(91.5) | 1787(90.0) | 1749(87.5) | 1697(85.1) | <0.001 |
| Depression,n(%) | 1016(50.9) | 934(47.0) | 903(45.2) | 890(44.6) | <0.001 |
| Sleep problems,n(%) | 1016(50.9) | 972(48.9) | 995(49.8) | 987(49.5) | 0.674 |
| Educational level,n(%) |  |  |  |  | 0.222 |
| No completion of primary school | 976(48.8) | 921(46.4) | 945(47.3) | 983(49.3) |  |
| Sishu/home school/elementary school | 433(21.7) | 449(22.6) | 465(23.3) | 453(22.7) |  |
| Middle school | 385(19.3) | 429(21.6) | 382(19.1) | 388(19.5) |  |
| High school and above | 204(10.2) | 187(9.4) | 206(10.3) | 170(8.5) |  |
| Smoking status,n(%) |  |  |  |  | 0.332 |
| Never | 1242(62.2) | 1220(61.4) | 1198(60.0) | 1190(59.7) |  |
| Quit | 146(7.3) | 162(8.2) | 181(9.1) | 158(7.9) |  |
| Still | 610(30.5) | 604(30.4) | 619(31.0) | 646(32.4) |  |
| Drinking status,n(%) |  |  |  |  | 0.053 |
| Never | 1221(61.1) | 1257(63.3) | 1190(59.6) | 1172(58.8) |  |
| Quit | 177(8.9) | 158(8.0) | 168(8.4) | 196(9.8) |  |
| Still | 600(30.0) | 571(28.8) | 640(32.0) | 626(31.4) |  |

**Table S4. The relationship between TyG-WHtR and the incidence of CVD was analyzed using multivariate Cox regression models**

|  | ModelⅠ | *P*-value | ModelⅡ | *P*-value | ModelⅢ | *P*-value |
| --- | --- | --- | --- | --- | --- | --- |
|  | HR (95% CI) |  | HR (95% CI) |  | HR (95% CI) |  |
| TyG-WHtR (per unit) | 1.426 (1.335-1.525) | <0.001 | 1.449 (1.322-1.588) | <0.001 | 1.446 (1.314-1.591) | <0.001 |
| TyG-WHtR (per SD) | 1.310 (1.251-1.378) | <0.001 | 1.326 (1.237-1.423) | <0.001 | 1.312 (1.224-1.453) | <0.001 |
| TyG-WHtR Q1 | Ref |  | Ref |  | Ref |  |
| TyG-WHtR Q2 | 1.254 (1.054-1.491) | 0.011 | 1.246 (1.043-1.488) | 0.015 | 1.235 (1.034-1.475) | 0.02 |
| TyG-WHtR Q3 | 1.606 (1.362-1.895) | <0.001 | 1.562 (1.308-1.865) | <0.001 | 1.565 (1.310-1.870) | <0.001 |
| TyG-WHtR Q4 | 2.029 (1.731-2.379) | <0.001 | 1.918 (1.578-2.332) | <0.001 | 1.883 (1.544-2.296) | <0.001 |

ModelⅠ: Crude model；

ModelⅡ: Adjusted for Age, Gender, HGB, PLT, BUN, Scr, UA, TC, HDL-c, LDL-c;

ModelⅢ: Adjusted for Age, Gender, HGB, PLT, BUN, Scr, UA, TC, HDL-c, LDL-c, Diabetes, Cancer, Lung disease, Liver disease, Education level, Marital status, Depression, Sleep problems, Smoking statues, Drinking statues.

**Table S5. The relationship between MAP and the incidence of CVD was analyzed using multivariate Cox regression models**

|  | ModelⅠ | *P*-value | ModelⅡ | *P*-value | ModelⅢ | *P*-value |
| --- | --- | --- | --- | --- | --- | --- |
|  | HR (95% CI) |  | HR (95% CI) |  | HR (95% CI) |  |
| MAP (per unit) | 1.017 (1.013-1.021) | <0.001 | 1.014 (1.010-1.018) | <0.001 | 1.015 (1.011-1.019) | <0.001 |
| MAP (per SD) | 1.270 (1.201-1.342) | <0.001 | 1.225 (1.151-1.277) | <0.001 | 1.246 (1.174-1.323) | <0.001 |
| MAP Q1 | Ref |  | Ref |  | Ref |  |
| MAP Q2 | 1.124 (0.949-1.331) | 0.177 | 1.081 (0.912-1.281) | 0.369 | 1.089 (0.919-1.291) | 0.323 |
| MAP Q3 | 1.466 (1.249-1.721) | <0.001 | 1.341 (1.140-1.577) | <0.001 | 1.364 (1.159-1.604) | <0.001 |
| MAP Q4 | 1.810 (1.551-2.114) | <0.001 | 1.576 (1.344-1.848) | <0.001 | 1.635 (1.393-1.919) | <0.001 |

ModelⅠ: Crude model；

ModelⅡ: Adjusted for Age, Gender, HGB, PLT, BUN, Scr, UA, TC, HDL-c, LDL-c;

ModelⅢ: Adjusted for Age, Gender, HGB, PLT, BUN, Scr, UA, TC, HDL-c, LDL-c, Diabetes, Cancer, Lung disease, Liver disease, Education level, Marital status, Depression, Sleep problems, Smoking statues, Drinking statues.

**Table S6. Associations of the TyG-WHtR index and MAP with the risk of CVD according to the quartiles of TyG-WHtR index and MAP**

|  | ModelⅠ | *P*-value | ModelⅡ | *P*-value | ModelⅢ | *P*-value |
| --- | --- | --- | --- | --- | --- | --- |
|  | HR (95% CI) |  | HR (95% CI) |  | HR (95% CI) |  |
| TyG-WHtRQ1 & MAPQ1 | Ref |  | Ref |  | Ref |  |
| TyG-WHtRQ1 & MAPQ2 | 0.814 (0.565-1.172) | 0.269 | 0.792 (0.550-1.142) | 0.212 | 0.774 (0.537-1.115 | 0.169 |
| TyG-WHtRQ1 & MAPQ3 | 1.241 (0.878-1.754) | 0.221 | 1.186 (0.838-1.677) | 0.336 | 1.203 (0.850-1.702) | 0.297 |
| TyG-WHtRQ1 & MAPQ4 | 1.578 (1.110-2.243) | 0.011 | 1.443 (1.013-2.053) | 0.042 | 1.506 (1.058-2.145) | 0.023 |
| TyG-WHtRQ2 & MAPQ1 | 1.020 (0.732-1.420) | 0.909 | 0.999 (0.716-1.395) | 0.995 | 0.979 (0.701-1.367) | 0.899 |
| TyG-WHtRQ2 & MAPQ2 | 1.189 (0.859-1.645) | 0.297 | 1.190 (0.858-1.650) | 0.298 | 1.188 (0.856-1.648) | 0.304 |
| TyG-WHtRQ2 & MAPQ3 | 1.561 (1.141-2.137) | 0.005 | 1.472 (1.073-2.020) | 0.017 | 1.459 (1.063-2.003) | 0.020 |
| TyG-WHtRQ2 & MAPQ4 | 1.966 (1.429-2.705) | <0.001 | 1.774 (1.285-2.449) | <0.001 | 1.804 (1.306-2.491) | <0.001 |
| TyG-WHtRQ3 & MAPQ1 | 1.547 (1.121-2.136) | 0.008 | 1.531 (1.102-2.128) | 0.011 | 1.479 (1.063-2.056) | 0.020 |
| TyG-WHtRQ3 & MAPQ2 | 1.530 (1.120-2.091) | 0.008 | 1.469 (1.068-2.021) | 0.018 | 1.493 (1.084-2.056) | 0.014 |
| TyG-WHtRQ3 & MAPQ3 | 1.695 (1.249-2.300) | <0.001 | 1.548 (1.133-2.116) | 0.006 | 1.572 (1.150-2.150) | 0.005 |
| TyG-WHtRQ3 & MAPQ4 | 2.187 (1.644-2.909) | <0.001 | 1.995 (1.488-2.675) | <0.001 | 2.032 (1.514-2.727) | <0.001 |
| TyG-WHtRQ4 & MAPQ1 | 1.522 (1.051-2.205) | 0.026 | 1.479 (1.004-2.179) | 0.048 | 1.432 (0.971-2.112) | 0.070 |
| TyG-WHtRQ4 & MAPQ2 | 2.007 (1.476-2.729) | <0.001 | 1.871 (1.348-2.597) | <0.001 | 1.804 (1.297-2.508) | <0.001 |
| TyG-WHtRQ4 & MAPQ3 | 2.346 (1.775-3.102) | <0.001 | 2.151 (1.592-2.905) | <0.001 | 2.116 (1.563-2.866) | <0.001 |
| TyG-WHtRQ4 & MAPQ4 | 2.509 (1.921-3.276) | <0.001 | 2.228 (1.664-2.984) | <0.001 | 2.248 (1.674-3.018) | <0.001 |

ModelⅠ: Crude model；

ModelⅡ: Adjusted for Age, Gender, HGB, PLT, BUN, Scr, UA, TC, HDL-c, LDL-c;

ModelⅢ: Adjusted for Age, Gender, HGB, PLT, BUN, Scr, UA, TC, HDL-c, LDL-c, Diabetes, Cancer, Lung disease, Liver disease, Education level, Marital status, Depression, Sleep problems, Smoking statues, Drinking statues.

**Table S7. Collinearity Statistics**

| Variables | VIF |
| --- | --- |
| Age | 1.341 |
| Gender | 2.816 |
| MAP | 1.116 |
| TyG-WHtR | 2.037 |
| PLT | 1.056 |
| HGB | 1.229 |
| BUN | 1.192 |
| Scr | 1.468 |
| UA | 1.471 |
| TC | 3.885 |
| HDLc | 1.566 |
| LDLc | 3.284 |
| Diabetes | 1.096 |
| Education level | 1.233 |
| Marital Status | 1.131 |
| Depression | 1.242 |
| Sleep problems | 1.222 |
| Smoking statues | 1.901 |
| Drinking statues | 1.449 |

**Table S8. Baseline characteristics after excluding individuals with any missing values**

| Characteristics |  |  |  |  |
| --- | --- | --- | --- | --- |
| Participants,No | | | 7442 | |
| Age,years,mean(SD) | | | 58.54(9.51) | |
| Gender,Female,n(%) | | | 3961(53.22) | |
| SBP,mmHg,mean(SD) | | | 129.69(21.31) | |
| DBP,mmHg,mean(SD) | | | 75.42(12.08) | |
| MAP,mmHg,mean(SD) | | | 93.51(14.17) | |
| Waist,cm,mean(SD) | | | 84.83(9.78) | |
| BMI,kg/m^2^，mean(SD) | | | 23.30(3.52) | |
| WHtR,mean(SD) | | | 0.54(0.06) | |
| HGB,g/dL,mean(SD) | | | 14.35(2.23) | |
| PLT,10^9^/L,mean(SD) | | | 211.56(73.11) | |
| BUN,mg/dL,mean(SD) | | | 15.70(4.58) | |
| Scr,mg/dL,mean(SD) | | | 0.78(0.24) | |
| UA,mg/dL,mean(SD) | | | 4.42(1.24) | |
| Glucose,mg/dL,mean(SD) | | | 109.49(35.60) | |
| HBA1C,%,mean(SD) | | | 5.25(0.79) | |
| TC,mg/dL,mean(SD) | | | 192.95(37.99) | |
| TG,mg/dL,mean(SD) | | | 129.34(94.23) | |
| HDL-c,mg/dL,mean(SD) | | | 51.62(15.21) | |
| LDL-c,mg/dL,mean(SD) | | | 115.90(34.76) | |
| eGFR,ml/minute/1.73m^2^,mean(SD) | | | 109.32(28.69) | |
| TyG.mean(SD) | | | 8.66(0.65) | |
| TyG-WHtR,mean(SD) | | | 4.67(0.75) | |
| Hypertension,n(%) | | | 1228(16.50) | |
| Diabetes,n(%) | | | 339(4.55) | |
| Cancer,n(%) | | | 70(0.94) | |
| Lung Diseases,n(%) | | | 673(9.04) | |
| Liver Diseases,n(%) | | | 271(3.64) | |
| CVD,n(%) | | | 1246(16.74) | |
| Marriage,married,n(%) | | | 6636(89.17) | |
| Depression,n(%) | | | 3497(46.99) | |
| Sleep problems,n(%) | | | 3694(49.64) | |
| Educational level,n(%) | | |  | |
| No completion of primary school | | | 3529(47.42) | |
| Sishu/home school/elementary school | | | 1716(23.06) | |
| Middle school | | | 1495(20.09) | |
| High school and above | | | 702(9.43) | |
| Smoking status,n(%) | | |  | |
| Never | | | 4505(60.53) | |
| Quit | | | 610(8.20) | |
| Still | | | 2327(31.27) | |
| Drinking status,n(%) | | |  | |
| Never | | | 4499(60.45) | |
| Quit | | | 657(8.83) | |
| Still | | | 2286(30.72) | |

**Table S9. Association of the TyG-WHtR index and MAP with CVD incidence after excluding individuals with any missing value using multivariate Cox regression models**

|  | ModelⅠ | *P*-value | ModelⅡ | *P*-value | ModelⅢ | *P*-value |
| --- | --- | --- | --- | --- | --- | --- |
|  | HR (95% CI) |  | HR (95% CI) |  | HR (95% CI) |  |
| TyG-WHtR < median & MAP < median | Ref |  | Ref |  | Ref |  |
| TyG-WHtR < median & MAP ≥ median | 1.583 (1.324-1.894) | <0.001 | 1.489 (1.243-1.782) | <0.001 | 1.525 (1.273-1.826) | <0.001 |
| TyG-WHtR ≥ median & MAP < median | 1.682 (1.413-2.003) | <0.001 | 1.552 (1.287-1.870) | <0.001 | 1.529 (1.266-1.845) | <0.001 |
| TyG-WHtR ≥ median & MAP ≥ median | 2.273 (1.947-2.652) | <0.001 | 1.951 (1.644-2.314) | <0.001 | 1.969 (1.656-2.342) | <0.001 |

ModelⅠ: Crude model；

ModelⅡ: Adjusted for Age, Gender, HGB, PLT, BUN, Scr, UA, TC, HDL-c, LDL-c;

ModelⅢ: Adjusted for Age, Gender, HGB, PLT, BUN, Scr, UA, TC, HDL-c, LDL-c, Diabetes, Cancer, Lung disease, Liver disease, Education level, Marital status, Depression, Sleep problems, Smoking statues, Drinking statues.

**Table S10. Demographic and clinical characteristics of the included cohort and excluded participants**

| Characteristics | included n=7976 | excluded n=9729 | P-value | SMD |
| --- | --- | --- | --- | --- |
|  |  |  |  |  |
| Age,years,mean(SD) | 58.68(9.60) | 59.36(10.58) | <0.001 | 0.067 |
| Gender,Female,n(%) | 4263(53.4) | 4965(51.1) | 0.002 | 0.046 |
| SBP,mmHg,mean(SD) | 129.82(21.35) | 131.67(20.95) | <0.001 | 0.088 |
| DBP,mmHg,mean(SD) | 75.46(12.08) | 76.57(12.17) | <0.001 | 0.092 |
| MAP,mmHg,mean(SD) | 93.58(14.17) | 94.94(14.02) | <0.001 | 0.097 |
| Waist,cm,mean(SD) | 84.82(9.80) | 84.89(10.62) | 0.680 | 0.007 |
| BMI,kg/m^2^，mean(SD) | 23.29(3.52) | 23.53(3.80) | <0.001 | 0.065 |
| WHtR,mean(SD) | 0.54(0.06) | 0.54(0.07) | <0.001 | 0.076 |
| HGB,g/dL,mean(SD) | 14.33(2.23) | 14.49(2.19) | <0.001 | 0.074 |
| PLT,10^9^/L,mean(SD) | 211.73(73.24) | 212.24(74.09) | 0.728 | 0.007 |
| BUN,mg/dL,mean(SD) | 15.72(4.57) | 15.81(4.80) | 0.299 | 0.021 |
| Scr,mg/dL,mean(SD) | 0.77(0.24) | 0.80(0.23) | <0.001 | 0.089 |
| UA,mg/dL,mean(SD) | 4.43(1.24) | 4.53(1.31) | <0.001 | 0.082 |
| Glucose,mg/dL,mean(SD) | 109.67(36.68) | 111.67(38.68) | 0.008 | 0.054 |
| HBA1C,%,mean(SD) | 5.25(0.80) | 5.29(0.86) | 0.019 | 0.047 |
| TC,mg/dL,mean(SD) | 193.23(38.83) | 192.39(39.02) | 0.277 | 0.022 |
| TG,mg/dL,mean(SD) | 131.96(109.87) | 138.73(94.90) | <0.001 | 0.064 |
| HDL-c,mg/dL,mean(SD) | 51.52(15.30) | 50.88(15.29) | 0.034 | 0.042 |
| LDL-c,mg/dL,mean(SD) | 115.96(34.98) | 116.07(37.76) | 0.873 | 0.003 |
| eGFR,ml/minute/1.73m^2^,mean(SD) | 109.22(29.17) | 108.21(30.78) | 0.088 | 0.034 |
| TyG.mean(SD) | 8.67(0.67) | 8.65(0.69) | 0.274 | 0.022 |
| TyG-WHtR,mean(SD) | 4.68(0.75) | 4.69(0.82) | 0.624 | 0.013 |
| Hypertension,n(%) | 1323(16.6) | 1701(17.8) | 0.041 | 0.032 |
| Diabetes,n(%) | 370(4.6) | 549(5.8) | 0.001 | 0.054 |
| Cancer,n(%) | 77(1.0) | 103(1.1) | 0.484 | 0.010 |
| Lung Diseases,n(%) | 729(9.2) | 1052(11.0) | <0.001 | 0.060 |
| Liver Diseases,n(%) | 286（3.6） | 390（4.1） | 0.105 | 0.026 |
| Marriage,married,n(%) | 7062（88.5） | 8361（86.1） | <0.001 | 0.072 |
| Depression,n(%) | 3644（47.1） | 3919（49.0） | 0.016 | 0.038 |
| Sleep problems,n(%) | 3918(49.8) | 4281(52.7) | <0.001 | 0.058 |
| Educational level,n(%) |  |  | 0.021 |  |
| No completion of primary school | 3825(48.0) | 4527(46.7) |  | 0.026 |
| Sishu/home school/elementary school | 1800(22.6) | 2100(21.7) |  | 0.022 |
| Middle school | 1584(19.9) | 2071(21.4) |  | 0.037 |
| High school and above | 767(9.6) | 996(10.3) |  | 0.023 |
| Smoking status,n(%) |  |  | 0.250 |  |
| Never | 4845(60.8) | 5719(59.6) |  | 0.025 |
| Quit | 647(8.1) | 823(8.6) |  | 0.018 |
| Still | 2476(31.1) | 3047(31.8) |  | 0.015 |
| Drinking status,n(%) |  |  | 0.017 |  |
| Never | 4835(60.7) | 5854(61.1) |  | 0.008 |
| Quit | 698(8.8) | 938(9.8) |  | 0.034 |
| Still | 2436(30.6) | 2790(29.1) |  | 0.033 |

**Table S11. Bonferroni correction was applied between groups**

|  | TyG-WHtR < median & MAP < median |  | TyG-WHtR < median & MAP ≥ median |  | TyG-WHtR ≥ median & MAP < median |  | TyG-WHtR ≥ median & MAP ≥ median |  |
| --- | --- | --- | --- | --- | --- | --- | --- | --- |
|  | χ² | *P*-value | χ² | *P*-value | χ² | *P*-value | χ² | *P*-value |
| TyG-WHtR < median & MAP < median |  |  | 26.4 | <0.001 | 34.378 | <0.001 | 115.513 | <0.001 |
| TyG-WHtR < median & MAP ≥ median | 26.4 | <0.001 |  |  | 0.354 | 0.552 | 21.414 | <0.001 |
| TyG-WHtR ≥ median & MAP < median | 34.378 | <0.001 | 0.354 | 0.552 |  |  | 16.321 | <0.001 |
| TyG-WHtR ≥ median & MAP ≥ median | 115.513 | <0.001 | 21.414 | <0.001 | 16.321 | <0.001 |  |  |

**Figure S1. KM plot of CVD based on the quartiles of the TyG-WHtR index and MAP.**


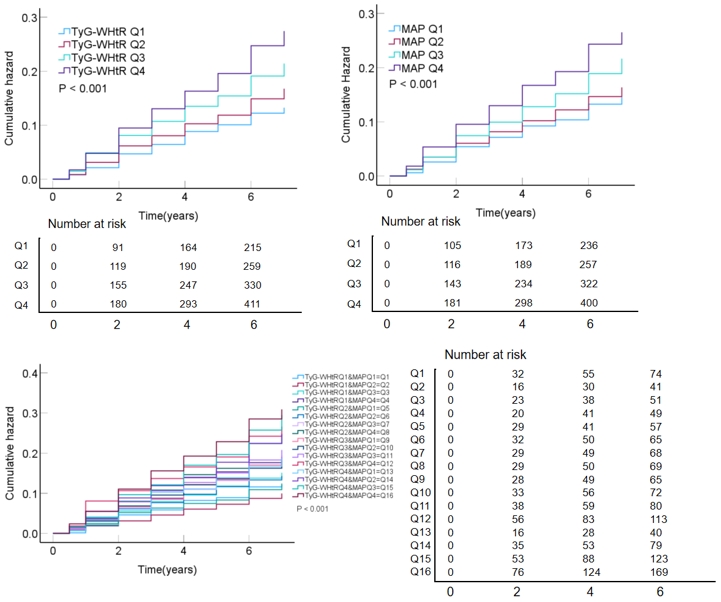


**Table S12. Subgroup analyses of the association of the TyG-WHtR and MAP with the risk of CVD.**

| Characteristics | HR (95% CI) | P-value | P for interaction |
| --- | --- | --- | --- |
| TyG-WHtR < median & MAP < median | Ref |  |  |
| Age |  |  | 0.001 |
| < 60 years |  |  |  |
| TyG-WHtR < median & MAP ≥ median | 1.822 (1.412-2.350) | <0.001 |  |
| TyG-WHtR ≥ median & MAP < median | 1.691 (1.306-2.189) | <0.001 |  |
| TyG-WHtR ≥ median & MAP ≥ median | 2.455 (1.930-3.122) | <0.001 |  |
| ≥ 60 years |  |  |  |
| TyG-WHtR < median & MAP ≥ median | 1.263 (0.996-1.602) | 0.054 |  |
| TyG-WHtR ≥ median & MAP < median | 1.385 (1.071-1.790) | 0.013 |  |
| TyG-WHtR ≥ median & MAP ≥ median | 1.618 (1.284-2.038) | <0.001 |  |
| Gender |  |  | 0.185 |
| Male |  |  |  |
| TyG-WHtR < median & MAP ≥ median | 1.624 (1.284-2.053) | <0.001 |  |
| TyG-WHtR ≥ median & MAP < median | 1.411 (1.035-1.924) | 0.03 |  |
| TyG-WHtR ≥ median & MAP ≥ median | 2.157 (1.682-2.766) | <0.001 |  |
| Female |  |  |  |
| TyG-WHtR < median & MAP ≥ median | 1.347 (1.035-1.753) | 0.027 |  |
| TyG-WHtR ≥ median & MAP < median | 1.520 (1.205-1.916) | <0.001 |  |
| TyG-WHtR ≥ median & MAP ≥ median | 1.782 (1.423-2.232) | <0.001 |  |
| Diabetes |  |  | 0.943 |
| Yes |  |  |  |
| TyG-WHtR < median & MAP ≥ median | 1.458 (0.377-5.638) | 0.584 |  |
| TyG-WHtR ≥ median & MAP < median | 1.195 (0.376-3.793) | 0.763 |  |
| TyG-WHtR ≥ median & MAP ≥ median | 1.563 (0.505-4.840) | 0.439 |  |
| No |  |  |  |
| TyG-WHtR < median & MAP ≥ median | 1.491 (1.251-1.778) | <0.001 |  |
| TyG-WHtR ≥ median & MAP < median | 1.564 (1.298-1.884) | <0.001 |  |
| TyG-WHtR ≥ median & MAP ≥ median | 1.960 (1.654-2.323) | <0.001 |  |
| Smoking status |  |  | 0.696 |
| Never |  |  |  |
| TyG-WHtR < median & MAP ≥ median | 1.303 (1.018-1.667) | 0.036 |  |
| TyG-WHtR ≥ median & MAP < median | 1.480 (1.181-1.855) | <0.001 |  |
| TyG-WHtR ≥ median & MAP ≥ median | 1.821 (1.469-2.257) | <0.001 |  |
| Quit |  |  |  |
| TyG-WHtR < median & MAP ≥ median | 1.382 (0.800-2.386) | 0.246 |  |
| TyG-WHtR ≥ median & MAP < median | 1.378 (0.758-2.505) | 0.293 |  |
| TyG-WHtR ≥ median & MAP ≥ median | 1.990 (1.188-3.333) | 0.009 |  |
| Still |  |  |  |
| TyG-WHtR < median & MAP ≥ median | 1.810 (1.366-2.399) | <0.001 |  |
| TyG-WHtR ≥ median & MAP < median | 1.670 (1.151-2.424) | 0.007 |  |
| TyG-WHtR ≥ median & MAP ≥ median | 2.188 (1.600-2.992) | <0.001 |  |
| Drinking status |  |  | 0.403 |
| Never |  |  |  |
| TyG-WHtR < median & MAP ≥ median | 1.351 (1.066-1.713) | 0.013 |  |
| TyG-WHtR ≥ median & MAP < median | 1.575 (1.258-1.972) | <0.001 |  |
| TyG-WHtR ≥ median & MAP ≥ median | 1.932 (1.563-2.389) | <0.001 |  |
| Quit |  |  |  |
| TyG-WHtR < median & MAP ≥ median | 1.906 (1.125-3.228) | 0.016 |  |
| TyG-WHtR ≥ median & MAP < median | 1.066 (0.563-2.021) | 0.844 |  |
| TyG-WHtR ≥ median & MAP ≥ median | 1.679 (0.968-2.914) | 0.065 |  |
| Still |  |  |  |
| TyG-WHtR < median & MAP ≥ median | 1.591 (1.179-2.146) | 0.002 |  |
| TyG-WHtR ≥ median & MAP < median | 1.536 (1.063-2.218) | 0.022 |  |
| TyG-WHtR ≥ median & MAP ≥ median | 1.986 (1.446-2.728) | <0.001 |  |

Age, Gender, HGB, PLT, BUN, Scr, UA, TC, HDL-c, LDL-c, Diabetes, Education level, Marital status, Depression, Sleep problems, Smoking statues, Drinking statues were adjusted, if not stratifed.

**Table S13. Four-way decomposition analyses of MAP in TyG-WHtR index and the incidence of CVD.**

| Effect | Estimate | Std.Error | CI_lower | CI_upper | P value |
| --- | --- | --- | --- | --- | --- |
| Controlled Direct Effect (CDE) | 0.389562 | 0.057281 | 0.280243 | 0.504010 | < 0.001 |
| Interaction Effect (INT) | -0.204748 | 0.130674 | -0.465253 | 0.026847 | 0.106 |
| Pure Indirect Effect (PIE) | 0.178300 | 0.073067 | 0.044593 | 0.322465 | 0.014 |
| Mediated Interaction (INTmed) | -0.023035 | 0.014697 | -0.051712 | 0.003100 | 0.106 |

Adjusted for Age, Gender, HGB, PLT, BUN, Scr, UA, TC, HDL-c, LDL-c, Diabetes, Cancer, Lung disease, Liver disease, Education level, Marital status, Depression, Sleep problems, Smoking statues, Drinking statues.

**Table S14. Four-way decomposition analyses of TyG-WHtR index in MAP and the incidence of CVD.**

| Effect | Estimate | Std.Error | CI_lower | CI_upper | P value |
| --- | --- | --- | --- | --- | --- |
| Controlled Direct Effect (CDE) | 0.013826 | 0.002349 | 0.009406 | 0.018541 | < 0.001 |
| Interaction Effect (INT) | -0.012542 | 0.007984 | -0.028726 | 0.001684 | 0.106 |
| Pure Indirect Effect (PIE) | 0.006475 | 0.002292 | 0.002269 | 0.010918 | 0.004 |
| Mediated Interaction (INTmed) | -0.000036 | 0.000023 | -0.000082 | 0.000005 | 0.106 |

Adjusted for Age, Gender, HGB, PLT, BUN, Scr, UA, TC, HDL-c, LDL-c, Diabetes, Cancer, Lung disease, Liver disease, Education level, Marital status, Depression, Sleep problems, Smoking statues, Drinking statues.
